# Multimodal imaging of the tricuspid valve: normal appearance and pathological entities

**DOI:** 10.1007/s13244-016-0504-7

**Published:** 2016-06-09

**Authors:** Soham Shah, Trevor Jenkins, Alan Markowitz, Robert Gilkeson, Prabhakar Rajiah

**Affiliations:** 1 Cardiothoracic Imaging, Department of Radiology, University Hospitals Case Medical Center, Cleveland, OH USA; 2 The Heart Valve Center, Harrington Heart and Vascular Institute, University Hospitals Case Medical Center, Cleveland, OH USA; 3 Cardiothoracic Imaging, Department of Radiology, UT Southwestern Medical Center, 5323 Harry Hines Boulevard, Dallas, TX 75390 USA

**Keywords:** Tricuspid valve, CT, MRI, Atrioventricular valve, Cardiac

## Abstract

The tricuspid valve, which is the atrioventricular valve attached to the morphological right ventricle, is affected by a wide range of pathological processes. Tricuspid valve diseases are now increasingly recognized as a significant cause of morbidity and mortality. Echocardiography is the most widely available and, hence, the first-line imaging modality used in the evaluation of tricuspid valve disorders; however, CT and MRI are also increasingly used for further evaluation and characterization of these entities. In this article, we first review the normal anatomy and embryology of the tricuspid valve, followed by a discussion of the role of multiple imaging modalities in the evaluation of tricuspid valve abnormalities. We then review and illustrate the imaging appearance of several congenital and acquired tricuspid valve abnormalities.

*Main Messages*

• *Tricuspid valve diseases have a significant impact on morbidity and mortality.*

• *CT and MRI are increasingly used in the evaluation of tricuspid disorders.*

• *CT and MRI help in diagnosis, functional evaluation, pre-surgical planning and post-surgical follow-up.*

• *The most common cause of tricuspid regurgitation is functional.*

**Electronic supplementary material:**

The online version of this article (doi:10.1007/s13244-016-0504-7) contains supplementary material, which is available to authorized users.

## Introduction

The tricuspid valve is affected by a wide variety of pathological entities, ranging from congenital abnormalities to neoplasms. Their impact also varies widely, from the characteristic stenosis and regurgitation to the more subtle carcinoid tumours and fibroelastomas. Because tricuspid valve and right heart abnormalities are usually asymptomatic, and are often secondary to left heart disorders, they have generally received less attention than the abnormalities on the left side [1]. However, recent studies have shown that tricuspid valve diseases have a significant impact on morbidity and mortality [2]. Tricuspid regurgitation may be asymptomatic for long periods, but severe tricuspid regurgitation may present with edema, anasarca, hepatic congestion, loss of appetite, fatigue, atrial fibrillation, abdominal pain, dyspnea, oliguria, ascites and jaundice.

Several imaging modalities are utilized in the evaluation of tricuspid valve disorders. Echocardiography is widely available, and is often the first-line imaging modality used for evaluating these disorders; however, computed tomography (CT) and magnetic resonance imaging (MRI) are also increasingly employed, not only for initial diagnoses and functional assessments, but also for pre-surgical planning to define complex anatomy, or even for post-surgical follow-up [3].

In this article, we first review the normal anatomy and embryology of the tricuspid valve, followed by a discussion of the role of multiple imaging modalities in the evaluation of tricuspid valve abnormalities. We then review and illustrate the imaging appearance of several congenital and acquired tricuspid valve abnormalities.

## Anatomy and physiology

**Fig. 1:**
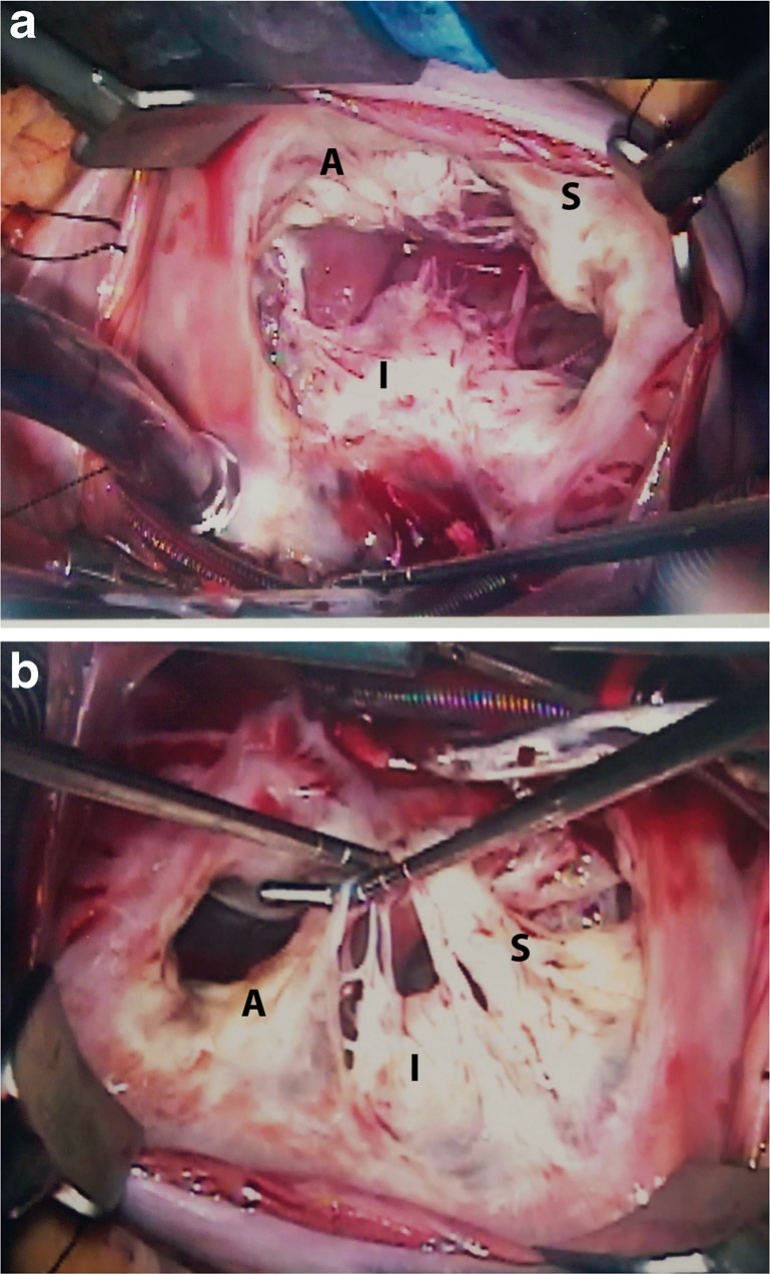
Normal surgical anatomy. (**a**) Superior view of the tricuspid valve shows the anterior leaflet (A), which is the largest; the septal leaflet (S), which is the smallest; and the posterior/inferior leaflet (P). (**b**) Another view of the tricuspid valve showing the papillary muscles, which are more numerous, smaller and more widely separated than those on the left side of the heart

The tricuspid valve is the atrioventricular (AV) valve that is attached to the morphological right ventricle (RV). The valve apparatus consists of three leaflets, chordae tendineae, two papillary muscles, fibrous annulus, and right atrial and ventricular myocardium [2]. The tricuspid valve is the most caudally located of all the cardiac valves and has the largest orifice [4]. The valve has three leaflets, namely the anterior (superior), inferior (posterior) and septal leaflets (Fig. 1a). The anterior leaflet is the largest and strongest, whereas the septal leaflet is the smallest, most medially located and the most immobile, directly originating from the fibrous annulus. The cusps are separated from each other by gaps of tissue referred to as commissures. The cusps have sturdy attachments to the annulus, since the collagenous matrix of each cusp fuses circumferentially with the fibrous annulus. The central fibrous body separates the tricuspid from the mitral valve. The endocardial portion at the peripheral edge of the valve fuses with the chordae tendineae, which in turn connects to the papillary muscles.

The papillary muscles of the RV (Fig. 1b) are smaller, more numerous and more widely separated than those of the left ventricle (LV). There are two sets of papillary muscles [2], namely the anterior and medial, with the anterior muscle providing chordae to the anterior and posterior leaflets, and the medial muscle providing chordae to the posterior and septal leaflets. The septal papillary muscle is either absent or diminutive. However, significant variability exists in the attachment of papillary muscles to the valve cusps through the chordae. The septal wall may provide chordae to the septal and anterior leaflets [2, 4]. Often, there are smaller chordae tendineae that attach the papillary muscles to the ventricular wall or function as bow strings between adjacent papillary muscles. Both the chordae tendineae and the septal leaflet of the tricuspid valve attach to the septum, which separates the right atrium (RA) from the LV. The tricuspid annulus, on which the leaflet sits, is a horseshoe-shaped saddle-like structure with an elliptical configuration. During right ventricular filling, the annulus opens and dilates more so along the free ventricular wall, becoming more spherical and ring-like.

Functionally, the tricuspid valve prevents retrograde flow of blood from the RV into the RA. The pulsatile nature of caval flow is caused by the cyclical opening and closing of the tricuspid valve. The valve opens with ventricular diastole and closes with ventricular systole.

## Embryology

The tricuspid valve apparatus is derived from the endothelial cells of the endocardial AV cushion tissue that separates the atria and ventricles and that contributes to the formation of the AV septum. The septal leaflet develops mainly from the inferior endocardial cushion, with a small contribution from the superior cushion. The leaflet subsequently delaminates from the myocardium. The anterior and posterior leaflets develop by invagination of a portion of the ventricular myocardium, including the lateral mesenchymal cushion, subepicardial mesenchyme and myocardium of the AV groove. This tunneling invagination process starts inferiorly and continues superiorly until the AV junction is reached. Thereafter, there is controlled resorption of the adjacent surrounding muscle tissue, which finally gives the valvular leaflets. This degenerative receding ventricular wall tissue also contributes to the chordae tendineae [5].

## Role of imaging modalities

Several imaging modalities are used in the evaluation of the tricuspid valve, particularly echocardiography, CT and MRI. Table 1 lists the advantages, disadvantages and features of these imaging modalities.

**Table 1:** Comparison of different imaging modalities used in the evaluation of the tricuspid valve

	Echocardiography	CT	MRI
Modes/sequences	• Gray scale	• Non-contrast	• Cine SSFP
	• 2D	• Contrast	• RV long-axis, RV horizontal long-axis, axial, short-axis, 4-chamber
	• 3D	• Static/dynamic	• Velocity-encoded phase contrast
	• Doppler		• Black-blood T1W, T2W, fat-saturated
			• Early contrast enhancement
			• Delayed contrast enhancement
Advantages	• Widely available	• Good spatial resolution	• Good spatial resolution
	• Low cost	• Good temporal resolution	• Good temporal resolution
	• Safe	• Multiplanar reconstruction capabilities	• Multiplanar imaging capabilities
	• Can be performed at bedside	• Calcification	• Morphological information
	• Can be performed even in hemodynamically unstable patients	• Evaluation of associated extracardiac disorders	• Tissue characterization
	• Morphological and functional evaluation	• Pre-surgical planning	• Accurate functional quantification of valve and ventricles
Disadvantages	• Operator-dependent	• Ionizing radiation	• Not widely available
	• Limited windows, especially for right heart	• Potentially nephrotoxic contrast media	• Higher cost
	• More limited in obesity, COPD, immediately post-surgery	• Dynamic evaluation/ventricular functional evaluation possible only in retrospective ECG-gated scans which is associated with higher radiation dose	• Time-consuming
	• Limited tissue characterization	• Valve function cannot be evaluated	• Contraindicated in some devices, claustrophobia
		• Limited in patients with high/irregular heart rates	• Calcifications not well seen
		• Limited tissue characterization	• Risk of nephrogenic systemic fibrosis in patients with severe renal dysfunction.
		• Only in hemodynamically stable patients	• Cannot be performed in hemodynamically unstable patients.
			• Requires breath-hold and steady heart rates

*SSFP* steady-state free precession, *RV* right ventricle, *T1W* T1-weighted, *T2W* T2-weighted

### Echocardiography

Because of its widespread availability and low cost, echocardiography is often the first-line modality for the evaluation of tricuspid valve pathologies. It can be performed safely even at the bedside in hemodynamically unstable patients. Echocardiography provides morphological information as well as functional evaluation of the tricuspid valve, using grayscale, 2D or 3D, and Doppler technique. However, echocardiography is operator-dependent, and may be limited in obese patients and those with emphysema, due to a restricted field of view.

### Computed tomography

CT is also valuable in the evaluation of the tricuspid valve, particularly for providing morphological information due to its good spatial and temporal resolutions and ability to do multiplanar reconstruction with isotropic resolution. Localization of a mass and its impact on the valve can be evaluated using CT. Calcifications are better evaluated with CT than MRI. Functional dynamic cine imaging can also be performed using retrospective ECG gating, albeit at a higher radiation dose. The scan protocol has to be optimized to ensure adequate contrast opacification around the valve and minimize artefacts. Typically, CT is performed using a prospective ECG-triggered acquisition to minimize motion and radiation, but retrospective ECG gating is chosen if cine images are required. Intravenous administration of 50–70 ml of contrast agent, followed by a 50/50 mixture of contrast and saline, is used to reduce streak artefacts from the superior vena cava (SVC). An alternate option is to use dual-route injection, with simultaneous injection of the upper and lower extremities. However, CT is associated with radiation and the use of potentially nephrotoxic contrast agents.

### Magnetic resonance imaging

MRI can provide comprehensive morphological and functional information on the tricuspid valve, with good spatiotemporal resolution, a large field of view and multiplanar imaging capabilities. MRI also provides functional assessment without the use of radiation and potentially nephrotoxic contrast media. It is ideal for evaluating the consequences of valvular abnormalities, such as the volume, mass and function of the ventricles. The tricuspid can be evaluated on MRI using steady-state free precession (SSFP) sequences in multiple planes, particularly the four-chamber, RV long-axis and short-axis views through the tricuspid annulus and valve. Right ventricular volumes can be measured in either the short-axis or axial plane, but the axial plane is associated with lower inter-observer variability [6]. Flow can be evaluated using a velocity-encoded flow quantification sequence. This can be measured either directly using short axis images through the tricuspid valve or indirectly from RV volumes of the short axis and forward flow obtained from the pulmonary artery. MRI also has superior tissue characterization capabilities, and lesions such as masses can be characterized using a combination of T1-weighted (T1W), T2-weighted (T2W) and fat saturation sequences and contrast enhancement, both early (perfusion) and delayed.

## Normal imaging appearance

**Fig. 2:**
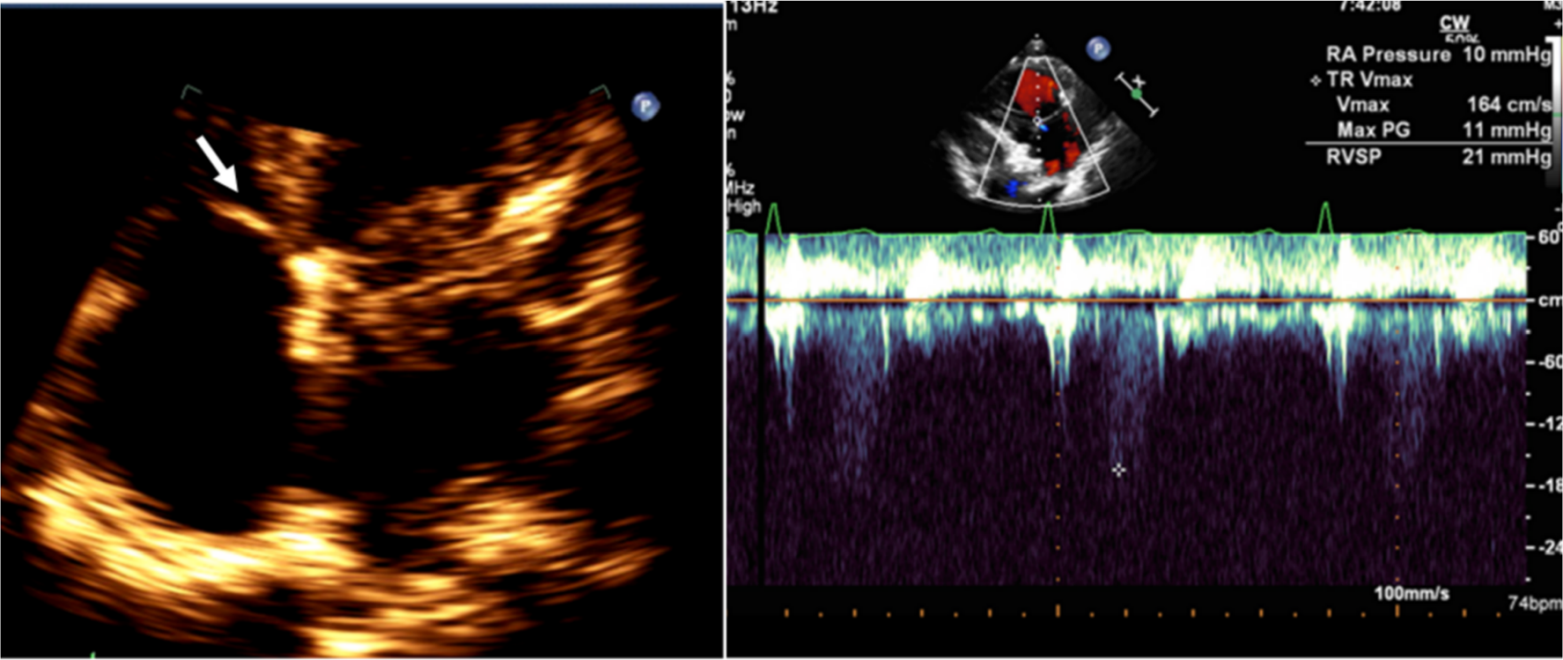
Normal echocardiographic appearance of tricuspid valve (*arrow*) and wave pattern

**Fig. 3:**
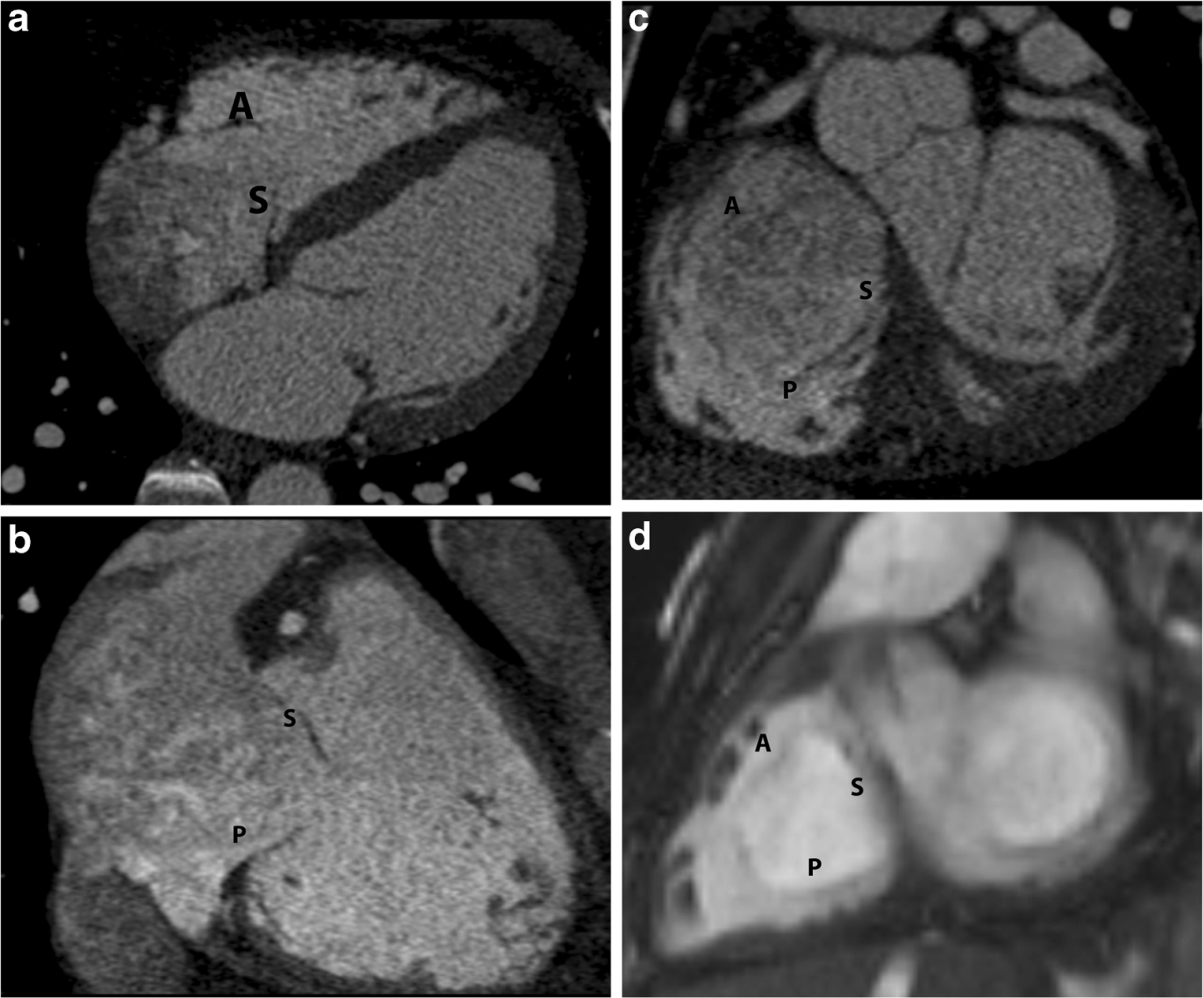
Normal appearance of tricuspid valve. (**a**) Four-chamber CT image shows the septal (S) and anterior (A) leaflets. (**b**) Two-chamber CT image shows septal (S) and posterior (P) leaflets. (**c**) Short-axis CT image shows all the leaflets (S, A, P). (**d**) MRI appearance of the tricuspid annulus, with the short-axis steady-state free precession (SSFP) image showing all the leaflets (S, A, P)

**Fig. 4:**
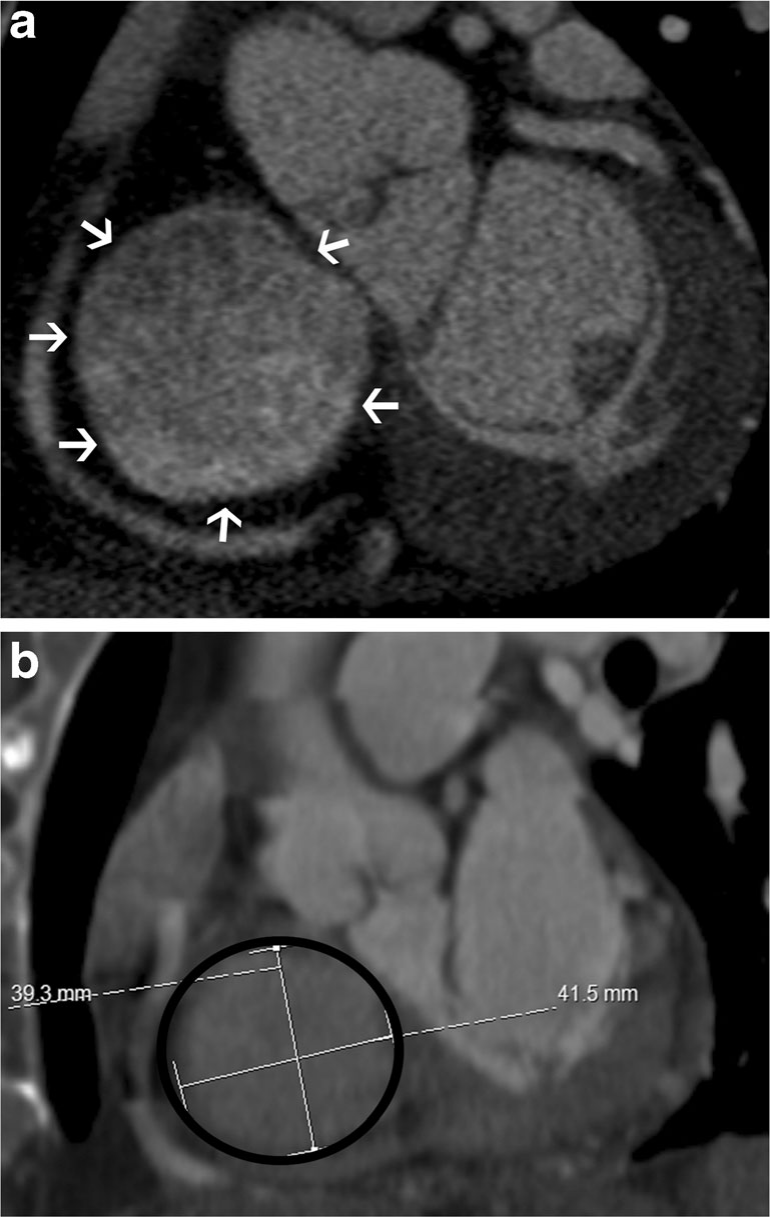
Tricuspid annulus. (**a**) Short-axis CT image of the tricuspid annulus,which is oval shaped and non-planar, with the septal leaflet attached in an oblique plane to the septum. The posterolateral is the lowest portion and the anteroseptal the highest portion of the annulus. (**b**) Measurement of the tricuspid annulus in CT, including circumference and diameters

On echocardiography, CT and MRI, a normal tricuspid valve is seen as a paper-thin structure (Figs. 2 and 3). The valve is non-planar, and hence the septal, anterior and posterior leaflets are not seen in the same plane (Fig. 3). The valve has an oval shape due to oblique attachment of the septal leaflet to the septum, with the posteroseptal portion being lower (towards the RV apex) than the anteroseptal portion. The septal leaflet is attached to the ventricular septum, and divides the membranous septum into interventricular and AV portions. The AV septum is located inferior to the membranous septum and this is the location of the bundle of His. Further inferiorly, the AV node is located just above the coronary sinus. The normal tricuspid annulus measures 30–35 mm in adults, i.e. 21 mm/m^2^ (Fig. 4). With ventricular systole, there is a reduction in annular size of up to 19 % in circumference and 30 % in area [7]. Compared to the mitral annulus, the tricuspid annulus is 20 % larger and is more apically located [4].

## Tricuspid valve dysfunction

Tricuspid valve dysfunction is seen in several primary and secondary abnormalities, both congenital and acquired. This can manifest as valvular stenosis, regurgitation or both.

### Tricuspid stenosis

**Fig. 5:**
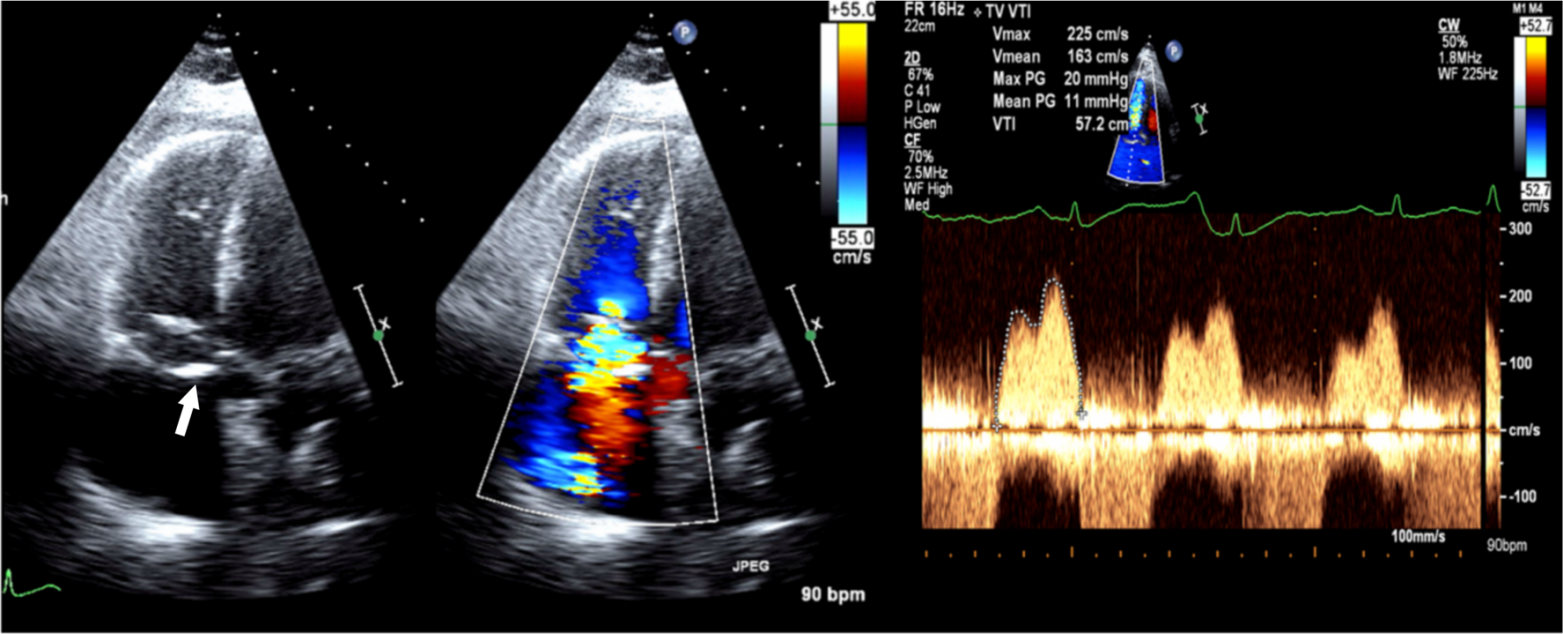
Tricuspid stenosis in echocardiogram. 2D and color Doppler images in a patient with anti-phospholipid antibody syndrome shows thickening of the valve leaflets (*arrow*) and continuous-wave Doppler with a mean pressure gradient of 11 mmHg, which is consistent with severe tricuspid stenosis

**Fig. 6:**
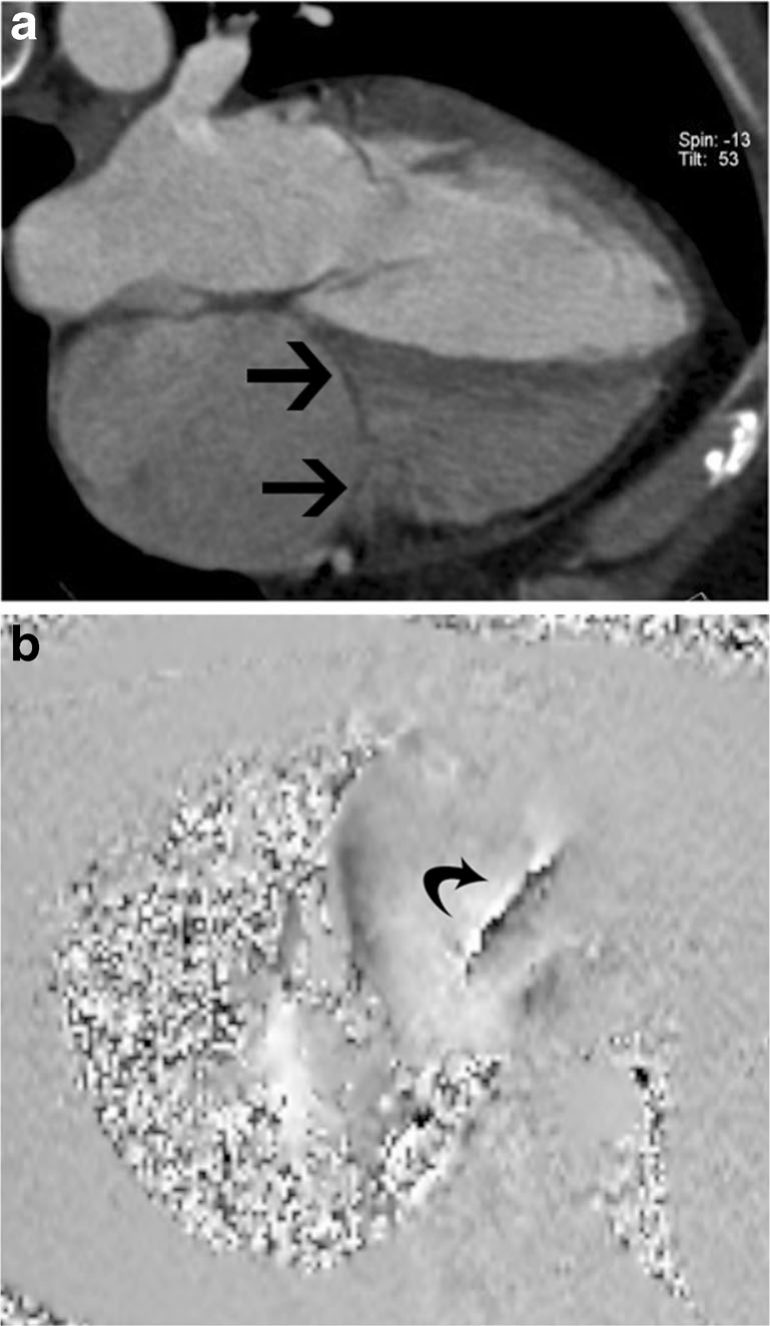
Tricuspid stenosis in CT and MRI. (**a**) Four-chamber reconstructed CT image shows thickening of the tricuspid leaflets (*arrows*) in a patient with carcinoid and tricuspid stenosis. (**b**) Four-chamber phase contrast velocity-encoded image shows a high-velocity jet extending across the tricuspid valve, resulting in aliasing (*arrow*)

Tricuspid stenosis is most commonly caused by rheumatic fever. Other causes include infective endocarditis, carcinoid, endomyocardial fibrosis, systemic lupus erythematosus (SLE), myxoma and congenital atresia. Rheumatic involvement of the tricuspid valve is not isolated and is usually associated with mitral and aortic valve involvement. It is also usually associated with tricuspid regurgitation [2]. Echocardiogram shows thick leaflets, limited leaflet mobility, decreased separation of leaflets, decreased annulus diameter, diastolic doming and increased velocity across the valve (Fig. 5). CT shows thickening and fusion of the leaflets (Fig. 6a), with or without calcification and right atrial dilatation (>20 cm^2^). On MRI, there is thickening of the leaflets with restricted diastolic opening and diastolic doming. There is restricted separation of commissures. The valve orifice, measured in short-axis images through the leaflet tips in diastole, < 1 cm^2^ is considered to be severe stenosis. The stenotic jet is seen as a signal void extending into the RV during diastole. The velocity of the stenosis can be quantified using velocity-encoded phase contrast imaging obtained along the plane (Fig. 6b) or perpendicular to the jet in the short axis. The peak gradient can be calculated using the modified Bernoulli equation, ∆P = 4V^2^, where ∆P is the pressure gradient and V is peak velocity. A mean pressure gradient > 5 mmHg is considered to be severe tricuspid stenosis [8]. Moderate or severe right atrial enlargement and dilated inferior vena cava (IVC) are also supportive features [8].

### Tricuspid regurgitation

Trace tricuspid regurgitation (TR) is seen in 80–90 % of healthy individuals. The most common cause (75 %) of pathological regurgitation is functional [7, 9], due to dilation of the annulus and valvular tenting, seen either due to RV dysfunction (left heart disease, ischemia, arrhythmogenic right ventricular dysplasia [ARVD], systemic RV in transposition of the great arteries [TGA)]), RV overload (pulmonary hypertension, systemic RV in TGA) or other causes (physiologic, atrial fibrillation, tumours). Left heart failure is the most common cause of TR. Structural TR is seen in 25 % and can either be congenital or acquired. Congenital causes include Ebstein’s anomaly, dysplasia, hypoplasia, cleft, prolapse, double orifice or bicuspid valve, whereas acquired causes include rheumatic fever, infective endocarditis, carcinoid, Loeffler endocarditis, trauma, tumours, or iatrogenic causes. Rheumatic fever is the most common cause of primary TR and is seen in 38 % of those with rheumatic mitral valve stenosis, with the valve appearing thickened and a with hockey stick-shaped appearance [2].

**Fig. 7:**
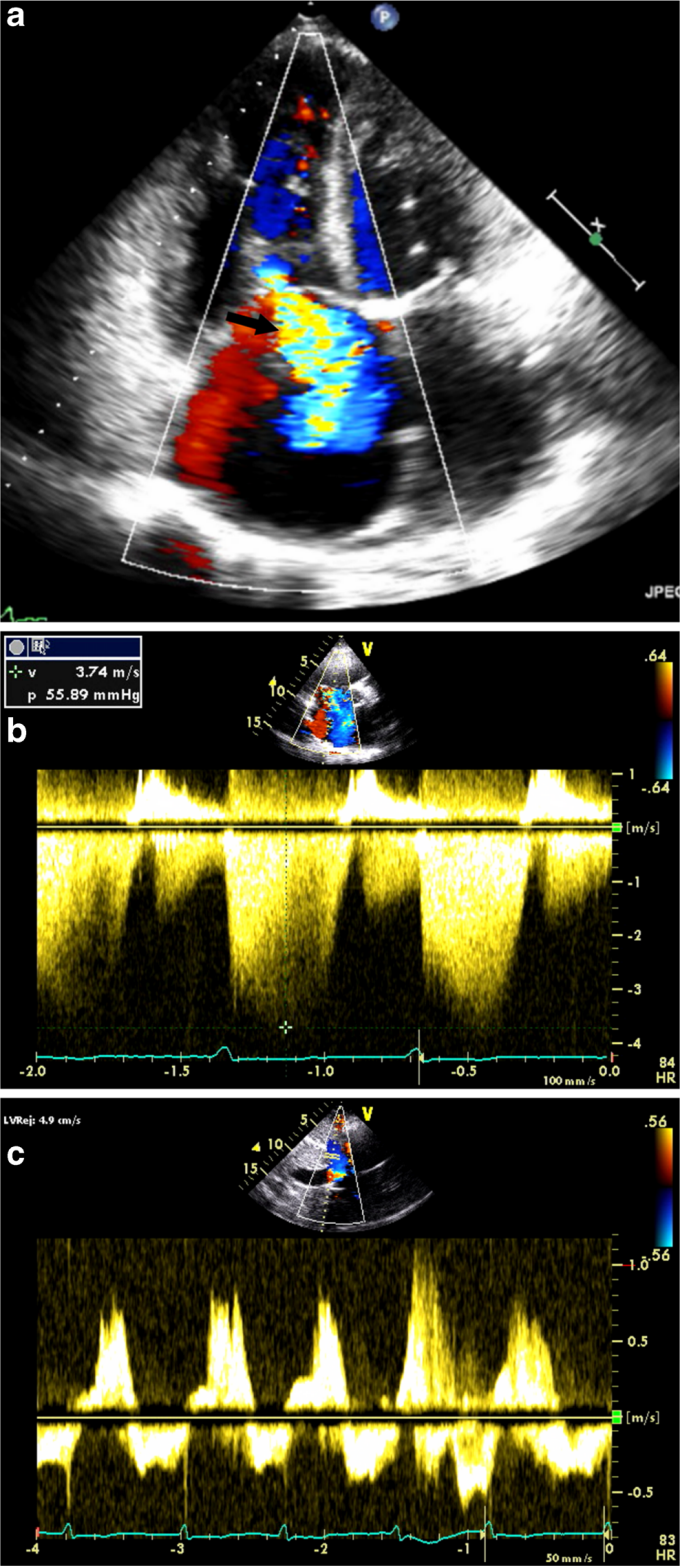
Echocardiography of tricuspid regurgitation (TR). (**a**) Apical four-chamber view with color Doppler demonstrates a markedly enlarged RA and a large TR jet (*arrow*) coursing along the interatrial septum. (**b**) Parasternal short-axis view with color Doppler demonstrates elevated RV pressure via peak TR jet velocity. (**c**) Hepatic vein continuous-wave Doppler with systolic reversal of flow in the setting of severe TR

**Fig. 8:**
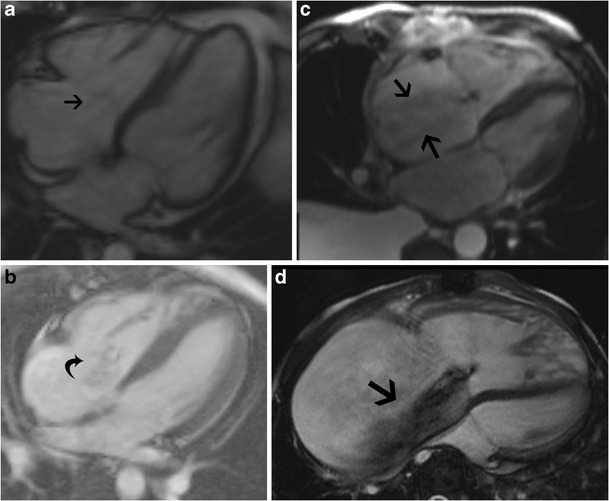
Grades of tricuspid regurgitation (TR). (**a**) Four-chamber steady-state free precession (SSFP) shows trace TR, extending just adjacent to the tricuspid valve (*arrow*). (**b**) Mild TR, with the jet extending just beyond the tricuspid valve into the proximal aspect of the right atrium (RA). (**c**) Moderate TR, with the jet extending to the middle third of the RA. (**d**) Severe TR, with the jet reaching the posterior wall of the RA

**Fig. 9:**
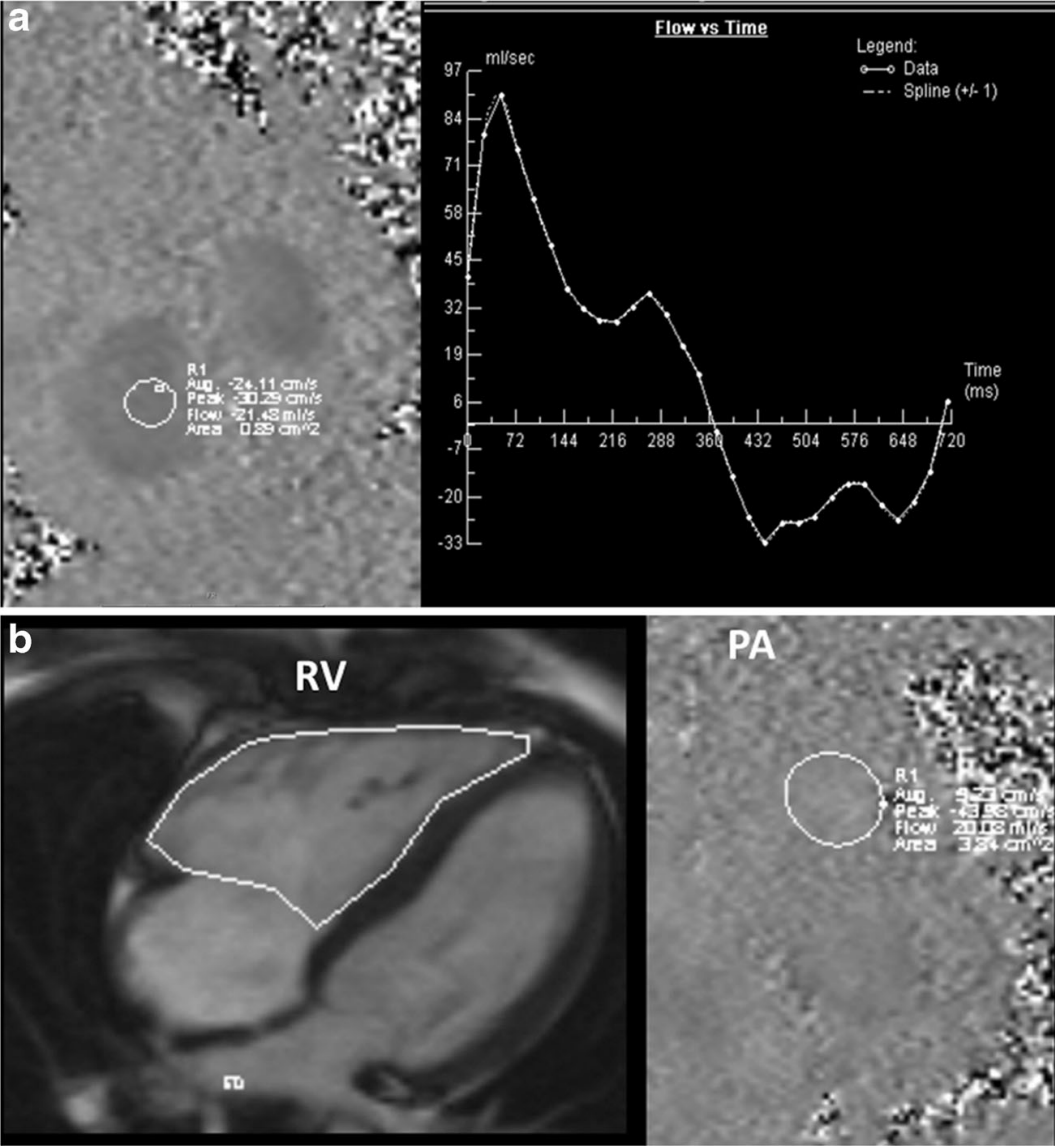
Quantification of tricuspid regurgitation (TR). (**a**) Direct measurement of TR using a velocity-encoded phase-contrast image obtained in the short-axis plane of the tricuspid valve. (**b**) Indirect measurement of TR using stroke volume from cine steady-state free precession (SSFP) and forward flow from the pulmonary artery

Echocardiography is the most commonly used modality in the evaluation of TR. The echocardiographic grades of TR are listed in Table 2 (Fig. 7). CT features of TR are dilated RA, RV, hepatic veins and IVC with systolic reflux of contrast into dilated hepatic veins and IVC. On MRI, a low signal regurgitant jet is seen extending through the tricuspid valve into the RA during systole. Visualization of the jet in the SSFP sequence may be difficult due to low shear and turbulence, but is appreciated in phase contrast velocity-encoded imaging. The jet can be qualitatively graded as mild, moderate and severe (Fig. 8) based on the extent of the jet posteriorly in the RA, jet area and jet area/left atrial area. Direct quantification of TR can be performed by performing the velocity-encoded sequence in the true short-axis plane of the tricuspid valve (Fig. 9a), but this is technically challenging due to extensive annular movement during the cardiac cycle [3]. TR can be indirectly quantified using stroke volumes obtained from RV volumetric analysis and pulmonary flow obtained from phase-contrast imaging. Tricuspid regurgitant fraction = (RV stroke volume − PA forward flow/RV stroke volume) x 100 % (Fig. 9b). Alternatively, (RV stroke volume − LV stroke volume)/RV stroke volume x 100 %. The accuracy of this technique is lower with arrhythmias

**Table 2:** Grades of tricuspid regurgitation in echocardiography [10]

	Mild	Moderate	Severe
Effective regurgitant orifice (mm^2^)	Undefined	Undefined	40
Regurgitation volume (ml)	Undefined	Undefined	>45
Vena contracta width (mm)	Undefined	<7	≥7
PISA radius (mm)	≤5	6–9	>9
Hepatic vein inflow	Systolic dominance	Systolic blunting	Systolic reversal
Tricuspid inflow	Normal	Normal	E-wave-dominant
Morphology	Normal/abnormal	Normal/abnormal	Abnormal/flail/large coaptation defect
Jet	Small, central	Moderate	Large central/eccentric and wall-impinging
Continuous-wave Doppler jet signal	Faint/parabolic	Dense/parabolic	Dense/triangular with early peaking

*PISA* proximal isovelocity surface area

**Fig. 10:**
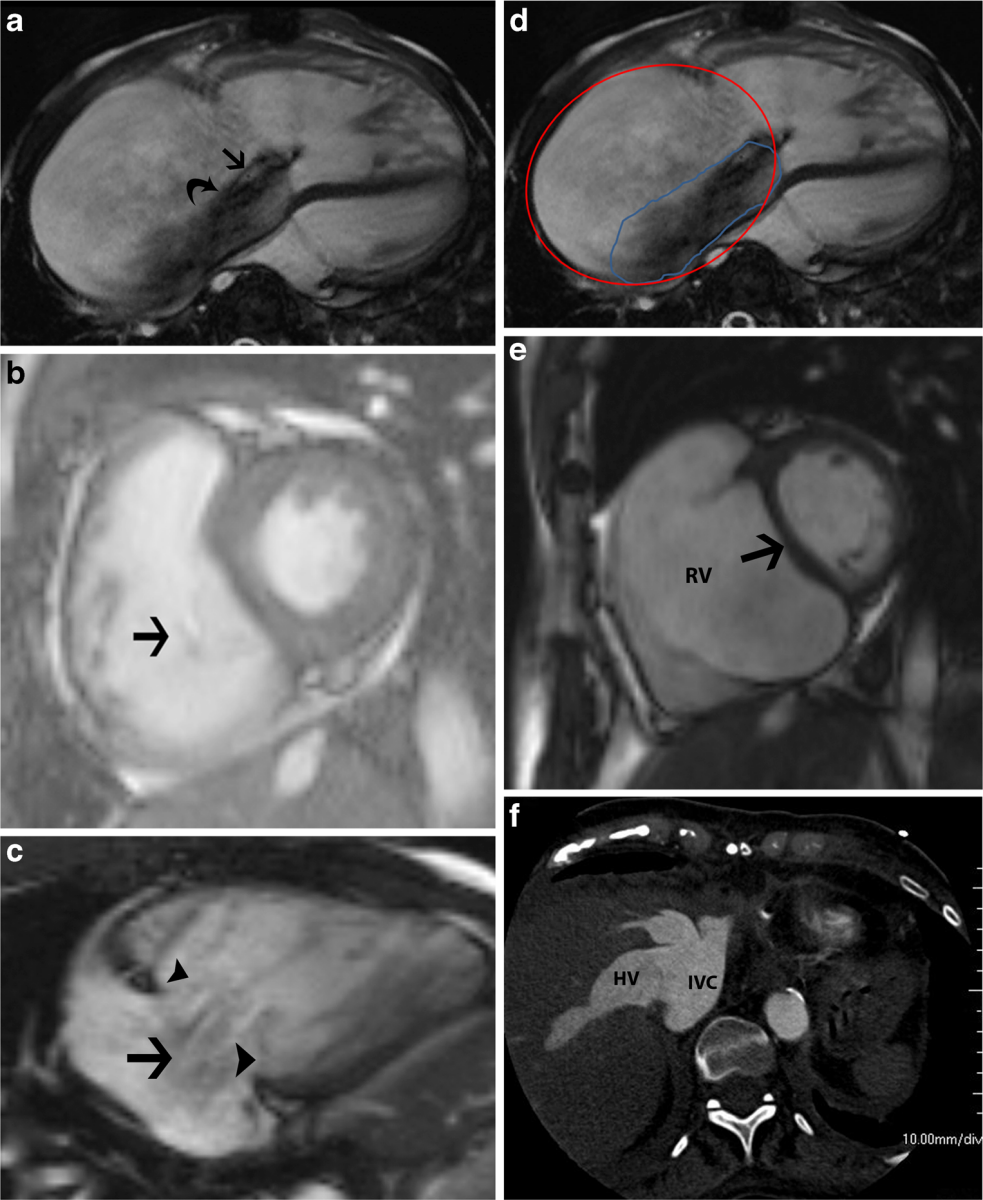
Quantification of tricuspid regurgitation (TR). (**a**) Vena contracta is the diameter of the smallest regurgitant flow (*straight arrow*) before expansion of the jet (*curved arrow*) in a four-chamber view. (**b**) Effective regurgitant orifice is measured from a short-axis cine steady-state free precession (SSFP) image through the tricuspid valve in systole. (**c**) Failure of coaptation of leaflets in four-chamber cine image (*arrowheads*) in a patient with significant TR (*arrow*). (**d**) Jet area (*blue*) and jet area/left atrial area are measured in a four-chamber SSFP or phase-contrast image. TR grade: mild > 5 cm^2^, moderate 6–10 cm^2^, severe >10 cm^2^. Ratio of regurgitant area to RA area: mild > 20 %, moderate 20–34 %, severe > 35 %. (**e**) Short-axis steady-state free precession (SSFP) image through the ventricles shows a flat ventricular septum (*arrow*). (**f**) CT image through the liver shows reflux of contrast through the hepatic veins and IVC

Other, less reliable techniques of quantification include measurement of the vena contracta, which is the smallest diameter of the regurgitant flow before the expansion of the jet as seen in the four-chamber view (Fig. 10a), and measurement of the effective regurgitant orifice (ERO) in short-axis views at the level of the leaflet tips (Fig. 10b). A vena contracta width > 7 mm is considered severe TR [10, 11]. Other features of severe TR include annular dilation > 40 mm, lack of coaptation of leaflets (Fig. 10c), jet area > 9 cm^2^, ratio of jet to RA area > 35 % (Fig. 10d), effective regurgitant orifice > 40 mm^2^, vena contracta > 7 mm, straight ventricular septum (Fig. 10e), atrial septal bulging, dilated RA, enlarged pulmonary artery, pulmonary regurgitation and systolic reversal in hepatic veins (Fig. 10f).

**Fig. 11:**
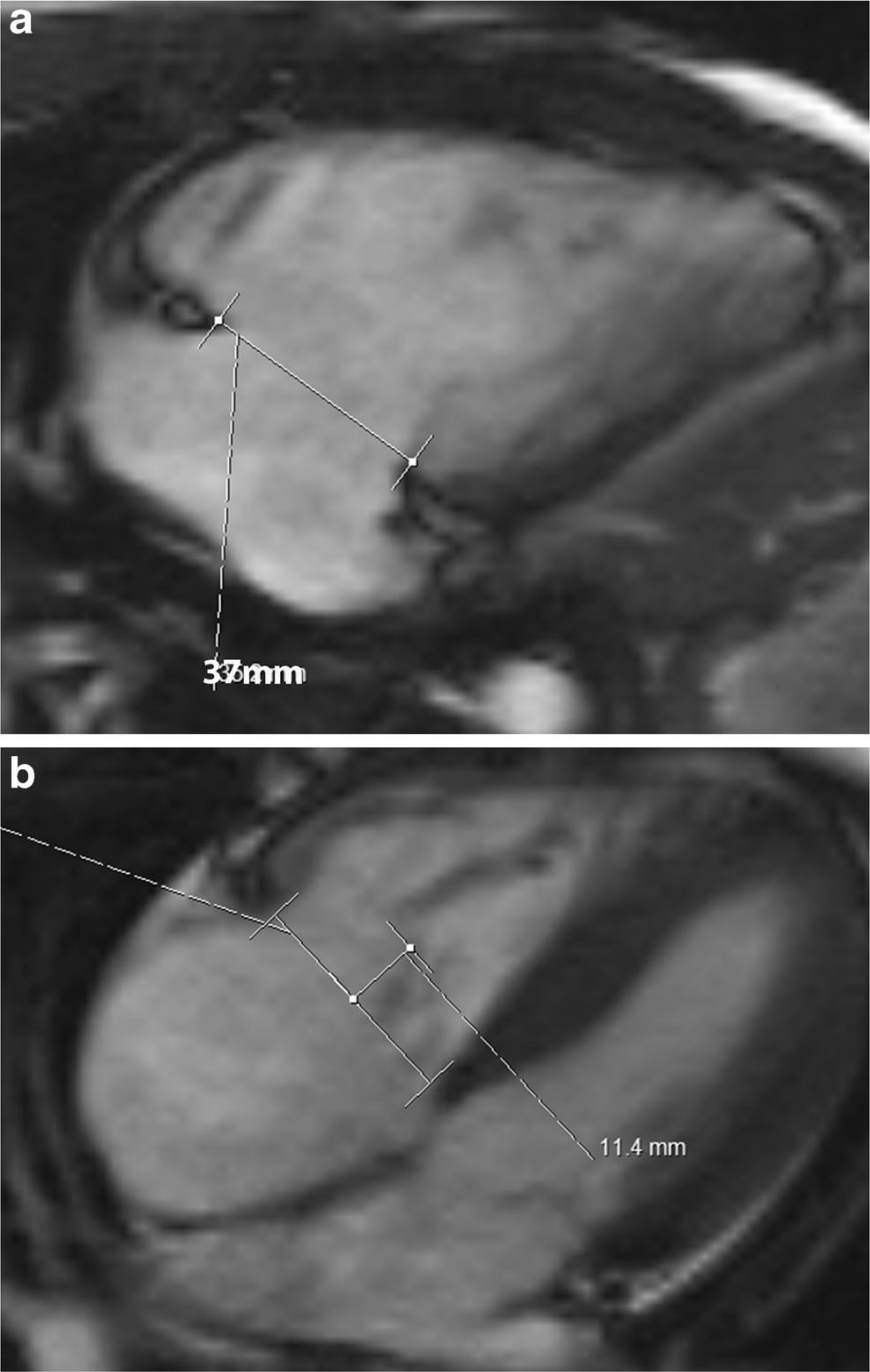
Tricuspid regurgitation (TR). (**a**) Four-chamber steady-state free precession (SSFP) image in a patient with functional TR shows dilation of the annulus > 35 mm. The annulus is also flat and planar. Dilation occurs along the free wall of the tricuspid annulus. The septal wall is intact. (**b**) Tethering. Four-chamber SSFP MRI image shows distance from the annular plane to coaptation in systole > 8 mm and area > 1.6 cm^2^, predicting residual TR following surgery

In functional TR, the annulus is dilated (>35 mm), planar, flat and circular (Fig. 11a). The annular dilation occurs on the free wall side, since the septal side is fixed. Also, the hinge points of the annulus are stretched away from the papillary muscles, resulting in tethering, which can be the only sign of TR, occurring occasionally even without valvular/annular dilation. Tenting distance is the distance between the annulus plane and valvular coaptation point (Fig. 11b). A tenting distance > 0.76 mm and tethering area > 1.63 cm^2^ indicate severe TR and predict residual TR after surgery [7]. The measurements are taken at end-expiration, and TR increases with inspiration. The normal systolic reduction of tricuspid annulus circumference/area is less prominent with functional TR, decreasing to 14.6 % [12].

**Fig. 12:**
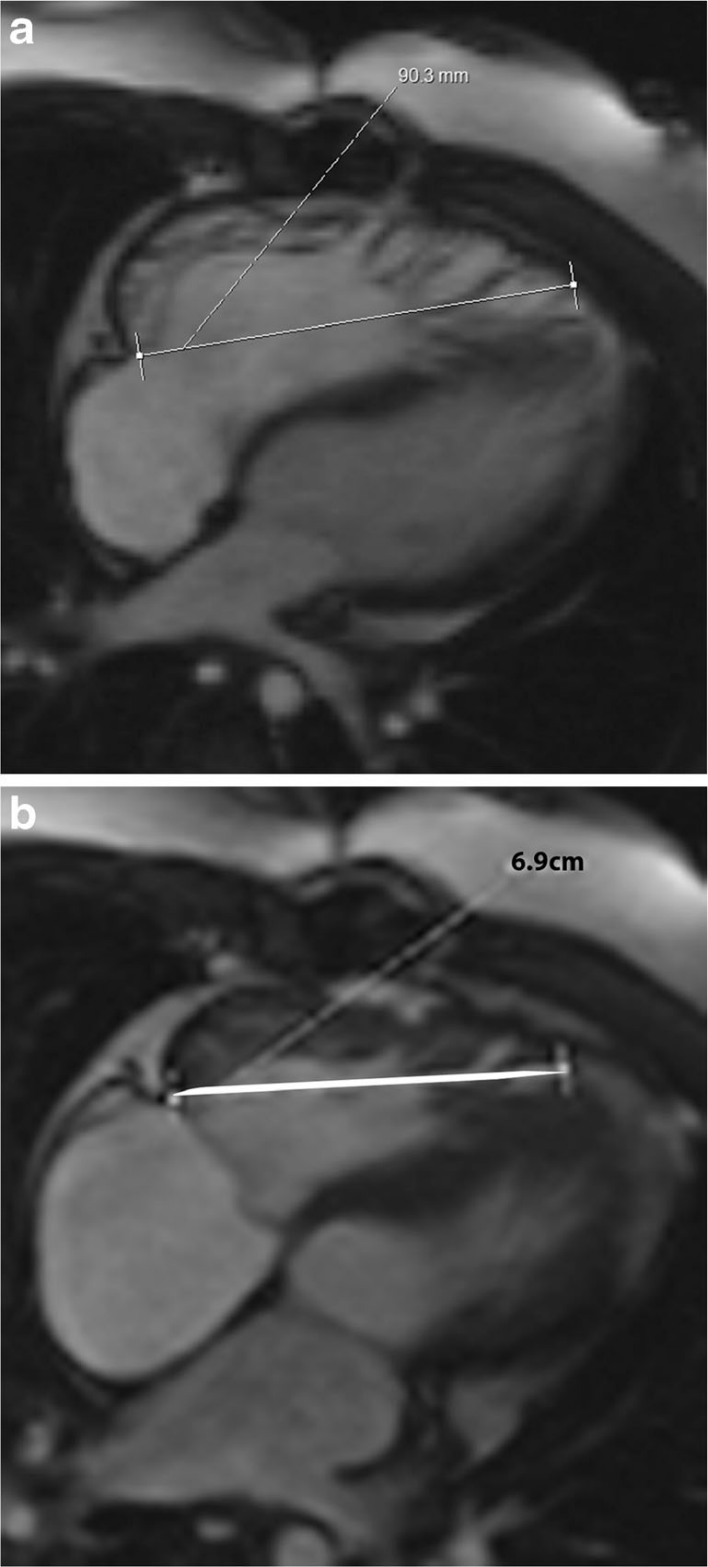
TAPSE. Tricuspid annular plane systolic excursion. (**a**) End-diastolic four-chamber steady-state free precession (SSFP) image shows the distance between the annulus and RV apex. (**b**) End-systolic four-chamber image in the same patient

MRI is considered the gold standard for the evaluation of RV volumes and function [13]. MRI measurement requires manual contouring of the RV endocardial border, both in end-diastole and end-systole. Tricuspid annular plane systolic excursion (TAPSE) is an increasingly used semi-quantitative measure of RV dysfunction which can be reliably obtained from cine SSFP MRI images in the four-chamber plane. MRI-TAPSE is the maximum apical excursion of the lateral tricuspid annular plane between end-diastole (Fig. 12 a) and end-systole (Fig. 12 b). The right ventricular ejection fraction (RVEF) is generally TAPSE multiplied by 2.5 [14]. A MR-TAPSE value below 20 mm indicates RV dysfunction [15]. High correlation has been shown between MR-TAPSE and RVEF in adult patients, both in normal and impaired RV function [15], but early results in children and young adults do not show correlation [16].

Surgery is performed with severe symptomatic TR or less severe TR with concomitant mitral valve disease, infective endocarditis, tetralogy of Fallot or annular diameter > 40 mm. If the functional TR is caused by annular dilation, it is treated by annuloplasty using rigid or flexible annular bands which decreases the annular size and restores the shape. If there is valvular tethering and RV dysfunction, a bioprosthetic or mechanical valve replacement is performed.

## Congenital conditions

### Tricuspid atresia

**Fig. 13:**
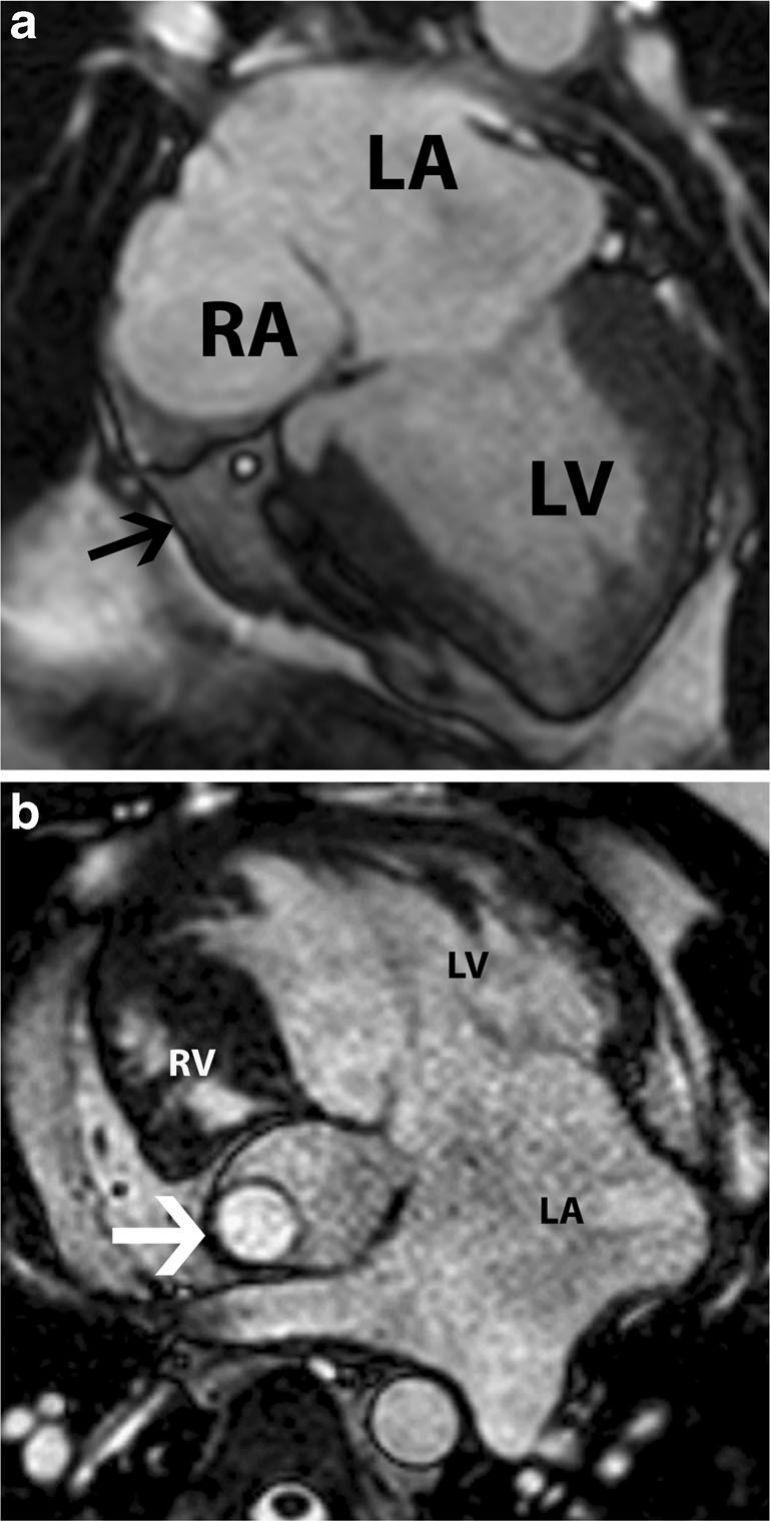
Tricuspid atresia. (**a**) Four-chamber steady-state free precession (SSFP) image shows absent tricuspid valve, which has been replaced by a fatty wedge of tissue in the right AV groove (*arrow*), a hypoplastic RV and dilated right atrium (RA). (**b**) Tricuspid atresia in a patient with dextro-transposition of the great arteries. The RV is hypoplastic. Note that this has been treated with a Fontan shunt (*arrow*)

Tricuspid atresia is the third most common congenital cyanotic heart disease, accounting for 2 % of all congenital cardiac disorders [17]. This is caused by embryological fusion of the dorsal and ventral endocardial cushions too far to the right lateral position. There is resultant obstruction to outflow from the RA to the RV; hence, both atrial septal defect (ASD) and ventricular septal defect (VSD) or a patent ductus arteriosus (PDA) are necessary for survival. There are three subtypes: I, normal great arterial arrangement (70 %; Fig. 13a); II, D-transposition of the great arteries (TGA; Fig. 13b); III, L-TGA [18].

On echocardiography, CT and MRI, the tricuspid valve is absent or abnormally closed. The areolar sulcal tissue occupying the gap where the AV connection and valve should have been present results in high signal in T1W and T2W MRI sequences. This distinguishes an absent AV connection from an imperforate valve. The RV is hypoplastic. Flow can be seen from the RA into the left atrium (LA) through the ASD and then through the mitral valve into the LV. The RA and systemic veins are dilated. All the types of tricuspid atresia are surgically treated by a three-stage repair with initial creation of an aortopulmonary shunt followed by the Glenn and Fontan procedure. Critical features for surgical management are the size of the VSD, ventriculoarterial concordance and pulmonary/subpulmonary stenosis/atresia.

### Ebstein’s anomaly

Ebstein’s anomaly is characterized by apical displacement of the tricuspid valve leaflets into the RV, which divides the RV into an ”atrialized” portion and a “functional” portion with intrinsic musculature, both of which show varying degrees of dysplasia, fibrosis and thinning. This anomaly is caused by failure of delamination of leaflets or incomplete extension medially of the valvular leaflet tissue from its origin from the ventricular wall. Ebstein’s anomaly primarily affects the septal and posterior leaflets of the tricuspid valve and their anomalous attachment to the valvular annular apparatus. The leaflets can be hypoplastic, aplastic, irregular or completely fused. The anterior leaflet may also be redundant, tethered, or exhibit fenestrations, or may be voluminous with a sail-like configuration. The tricuspid annulus has varying levels of rotation and obliquity. The chordae tendineae and papillary muscles have dysplasia and varying levels of myocardial insertion. Associated anomalies include patent foramen ovale (PFO), ASD and VSD.

**Fig. 14:**
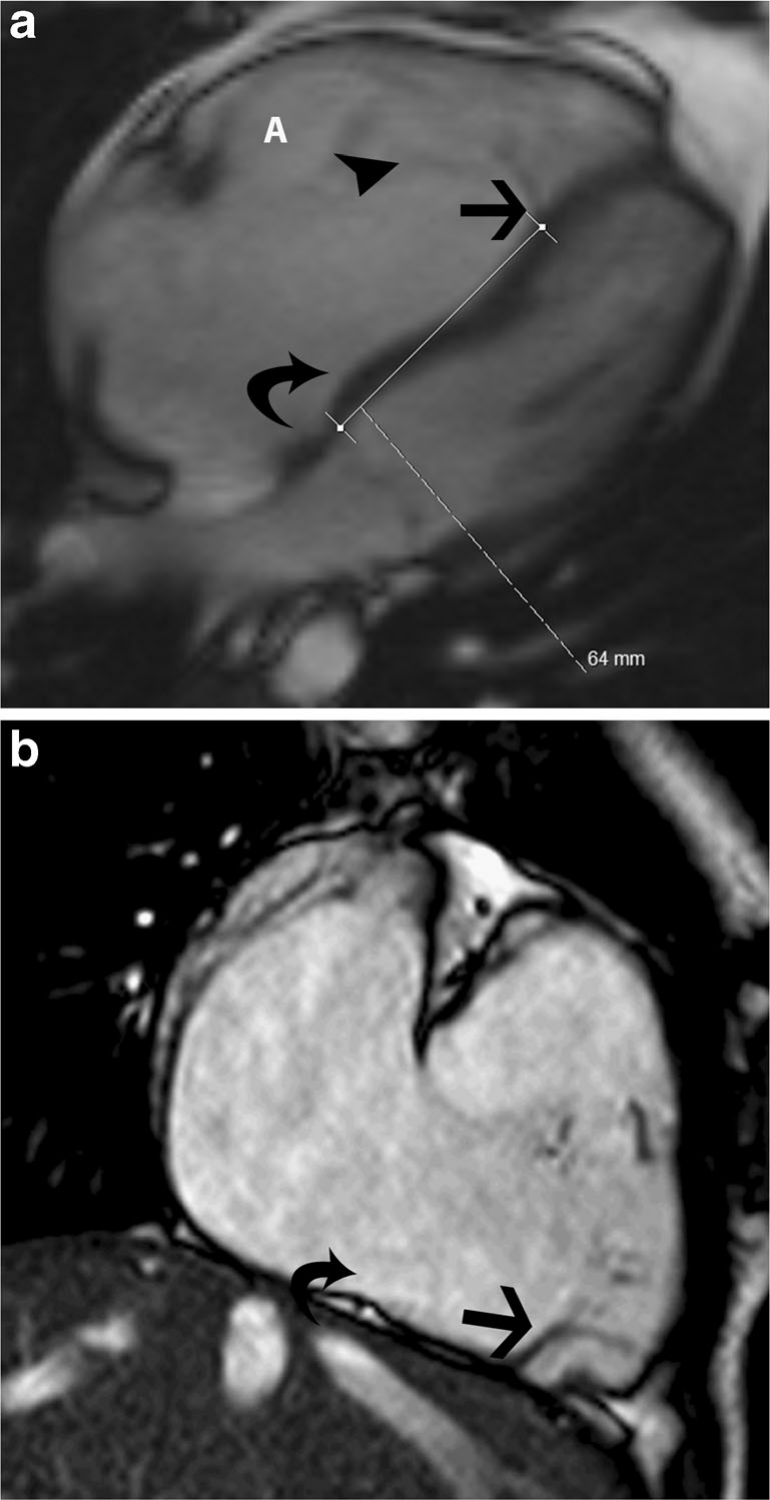
Ebstein’s anomaly. (**a**) Four-chamber steady-state free precession (SSFP) image shows significant apical displacement (64 mm) of the septal leaflet of the tricuspid valve *(straight arrow*) from the level of the tricuspid annular plane (*curved arrow*). Note that the anterior leaflet (*A*) is located normally. (**b**) Vertical long-axis SSFP image of the same patient shows significant apical displacement of the posterior leaflet of the tricuspid valve (*straight arrow*) from the level of the tricuspid annular plane (*curved arrow*)

On CT and MRI, Ebstein’s anomaly is diagnosed when there is apical displacement of either the septal or posterior tricuspid valve leaflet > 0.8 cm/m^2^ [19] from the level of the insertion point of the anterior mitral leaflet (Fig. 14, Movie S1). Other features include dilated anatomic right AV junction, leaflet thickening, wall thinning of the proximal “atrialized” portion of the RV, discontinuity of the annular ring apparatus with displacement and obliquity, TR, and varying levels of RV dysfunction. In severe or chronic manifestations of the disease, there are signs of RV failure such as paradoxical motion of the ventricular septum and bowing (Movie S1). Late gadolinium enhancement may be seen in the ventricular wall. In addition to providing morphological information about the valve apparatus and associated cardiovascular abnormalities, MRI provides functional information through quantification of ventricular, regurgitant and shunt volumes, which helps in pre-surgical planning. Sudden death may occur in Ebstein’s anomaly due to ventricular pre-excitation caused by accessory muscle pathways. Ebstein’s anomaly is treated with surgical correction.

### Aneurysm of the membranous ventricular septum

**Fig. 15:**
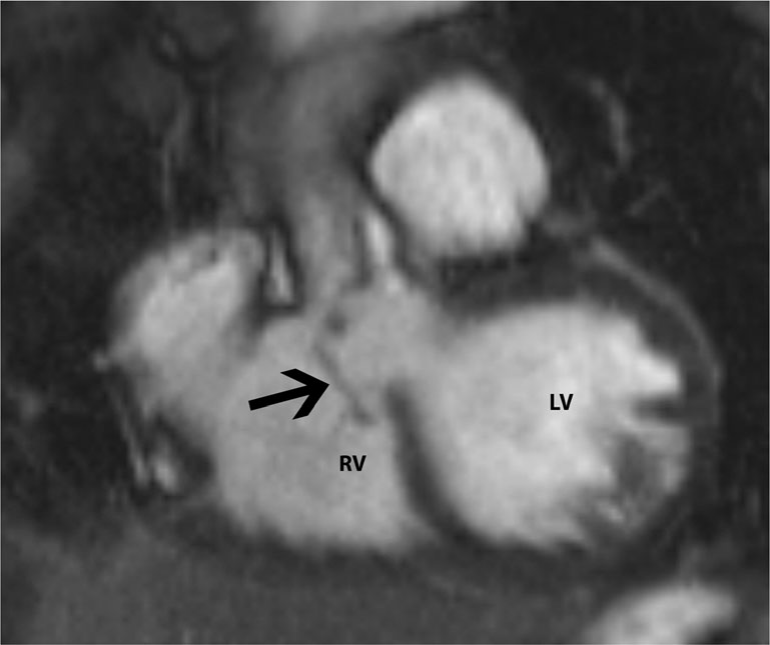
Aneurysm of the membranous septum. Coronal steady-state free precession (SSFP) MRI image shows an aneurysm of the membranous septum, formed by adhesion of the septal tricuspid leaflet to a rim of membranous ventricular septal defect (VSD). This results in partial or complete closure of the VSD with or without an aneurysm formation

Aneurysm of the membranous ventricular septum refers to a “windsock” type of aneurysm at the basal membranous ventricular septum. It is associated with VSD (in 19 %) [20] and other congenital defects. This is believed to be a sequel of a membranous ventricular septal defect that is either completely or partially closed by adhesion of the septal leaflet of the tricuspid valve to the rim of the membranous VSD. It is usually an incidental discovery in imaging studies. Complications include rupture, endocarditis, arrhythmia, thromboembolism and right- or left-ventricular outflow obstruction [20]. On CT and MRI, aneurysmal bulging of the membranous portion of the ventricular septum is noted (Fig. 15). It is important to distinguish this entity from a sinus of Valsalva aneurysm which is located superior to the plane of the left ventricular outflow tract. A VSD may be identified either morphologically or by demonstration of a shunt.

### Gerbode defect

**Fig. 16:**
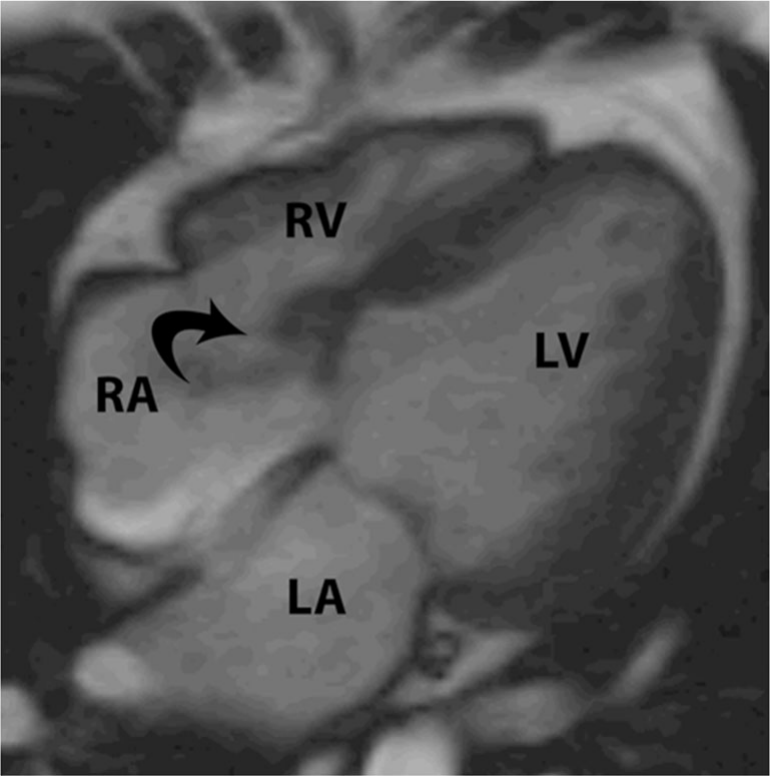
Gerbode defect. Four-chamber steady-state free precession (SSFP) image shows a defect and a shunt extending from the left ventricle (LV) to the right atrium (RA) through a Gerbode defect

A Gerbode defect is a ventriculoatrial defect, with communication between the LV and the RA. In the *congenital* type, there is a defect in the AV component of the membranous septum, resulting in direct shunting from the LV to the RA, above the hinge points of the tricuspid valve leaflets. In the *indirect type*, the shunting happens through a peri-membranous VSD into the RV below the level of the tricuspid valve, after which the atrial shunting happens through a cleft in the septal leaflet of the tricuspid valve [21] (Fig. 16, Movie S2). The *acquired* type of Gerbode defect is caused by trauma, iatrogenic surgery or infective endocarditis. Gerbode defects are treated with surgical closure using a patch.

### Transposition of the great arteries

**Fig. 17:**
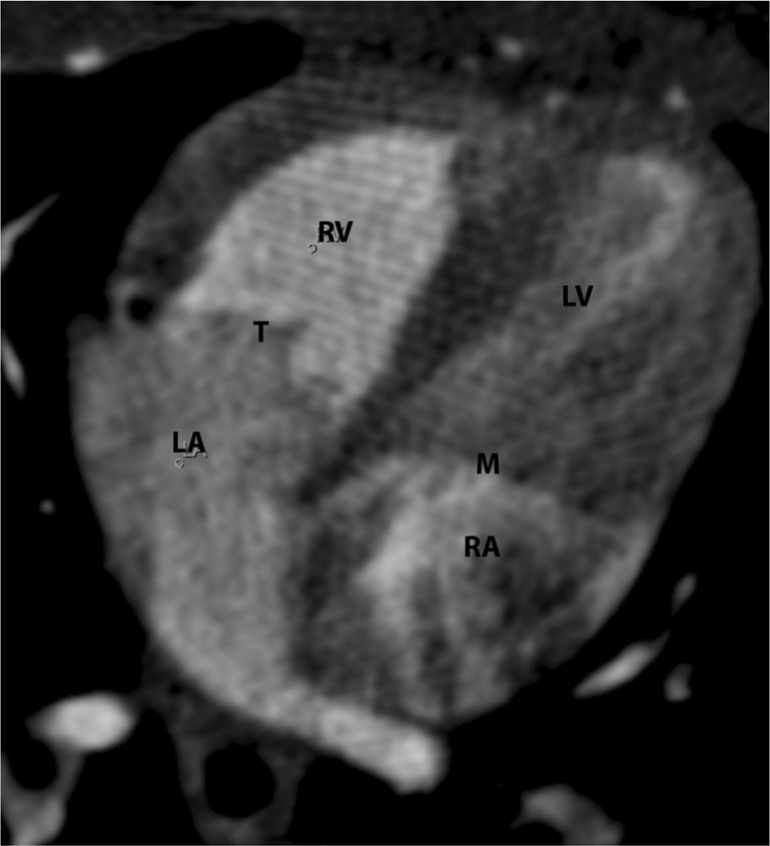
L-transposition of the great arteries. The tricuspid valve is the valve that is attached to the morphological right ventricle (RV). In this patient with L-TGA, where the RV is connected to the aorta (not shown here), the tricuspid valve (T) is attached to the RV. The left atrium (LA) drains into the RV

Transposition of the great arteries (TGA) refers to ventriculoarterial discordance, with or without AV discordance. Broadly there are two types, namely dextro-transposition (D-TGA), and levo-transposition (L-TGA). In both types, there is ventriculoarterial discordance, i.e., the morphological RV is connected to the aorta and the morphological LV is connected to the pulmonary artery. In L-TGA, there is also AV discordance, with the RA connected to the LV and the LA to the RV. In both abnormalities, the tricuspid valve is the valve that is attached to the morphological RV (Fig. 17). The RV is not necessarily the ventricle on the right side of the heart, but is defined by its structure. The morphological RV is distinguished from the morphological LV by the presence of septomarginal trabeculations, moderator band, > 3 papillary muscles and the absence of fibrous continuity between the AV and semilunar valves.

### AV canal defect

A complete AV canal defect (endocardial cushion defects, common AV canal) is a defect of the AV septum, with a common AV valve, atrial and ventricular septal defects. Embryologically, there is a failure of the AV junction to develop into two distinct AV valves, as well as failure to form atrial and ventricular septa. AV canal defects are seen in 40 % of Down syndrome patients, and 30 % of AV canal defects are associated with Down syndrome [22]. There is left-to-right shunting, depending on the size of the defect, with biventricular volume overload. Heart failure ensues if this is not surgically corrected in the first few months of life.

**Fig. 18:**
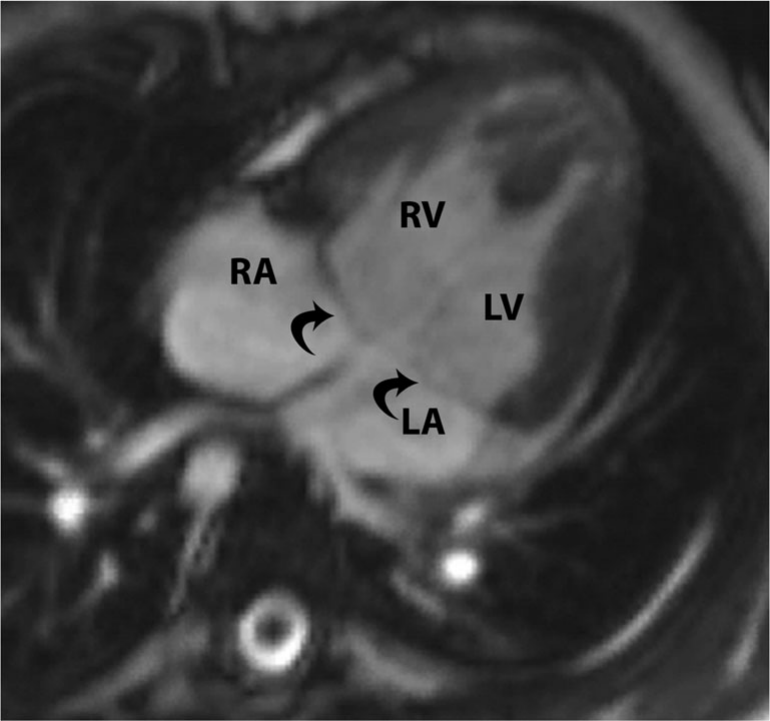
Atrioventricular (AV) canal defect. Four-chamber steady-state free precession (SSFP) image in a patient with AV canal defect shows a common AV valve (*straight arrow*)

On MRI, a common AV valve ring and valve is seen (Fig. 18). This is associated with ostium primum atrial septal and membranous ventricular septal defects. The common AV valve may be seen connecting to the ventricular septal crests by accessory chordae, although there is incomplete bridging. There is elongation, narrowing and crowding of the LV outflow tract, which is termed the “gooseneck deformity”, caused by the anterior displacement of the aorta and the main pulmonary artery as a result of the arrested formation of the AV canal [22]. Treatment involves patch closure of the ASD and VSD, as well as the creation of a “sling” to separate and suspend the valve leaflets along the surgically created septal attachments.

### Crisscross heart

**Fig. 19:**
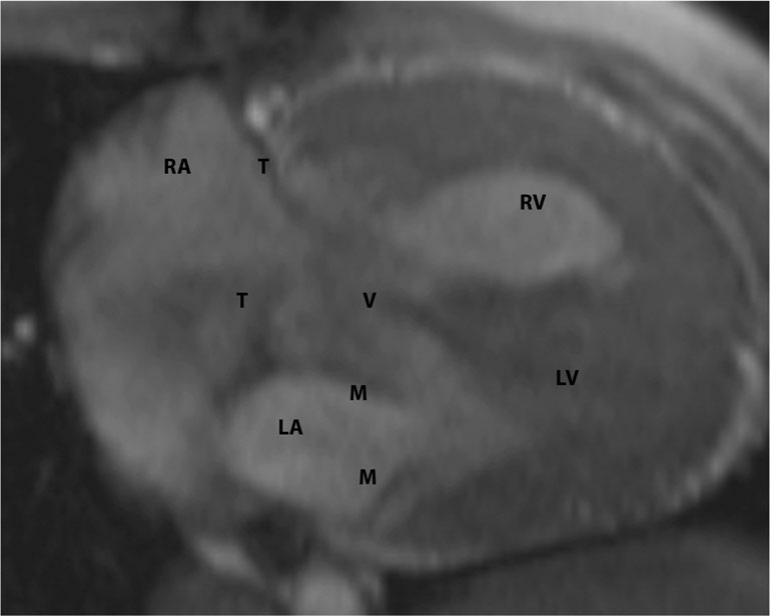
Crisscross heart. Four-chamber static steady-state free precession (SSFP) image shows crossing of the inflow streams of the tricuspid (T) and mitral (M) valves, due to twisting of the apex of the heart at its axis in a superoinferior configuration of ventricles. This gives the appearance of atria emptying into contralateral ventricles

Crisscross heart is a rare complex congenital malformation (0.1 %) in which there is a spatially discordant positioning of the RA with respect to the LV, and the LA with respect to the RV, although the functional connections are normal [23]]. The RA is connected to the RV, which is abnormally located where the LV is normally situated, and the LA is connected to the LV, which is abnormally located where the RV is normally located. There is also a discordant crisscross anomaly, where the RA is connected to the LV, with the LV located in its normal spatial position. In the third type, there is AV discordance with spatially discordant ventricles, with the RA connected to the LV, which is in the anatomic position of the RV. In addition, there is variability in the ventriculoarterial concordance, with both dextro- and levo-transposition of the great arteries possible. Embryologically, the ventricles twist around their longitudinal axis (with the atria remaining stationary) after the establishment of valvular AV connections. CT and MRI are valuable in the evaluation of the complex anatomy and connections of this abnormality and in the quantification of ventricular and valvular function and shunt fractions [23]. On MRI, the term "crisscrossing" is applied when there is crossing of the blood jet stream across the septum (Fig. 19).

## Acquired conditions

### Carcinoid tumour

**Fig. 20:**
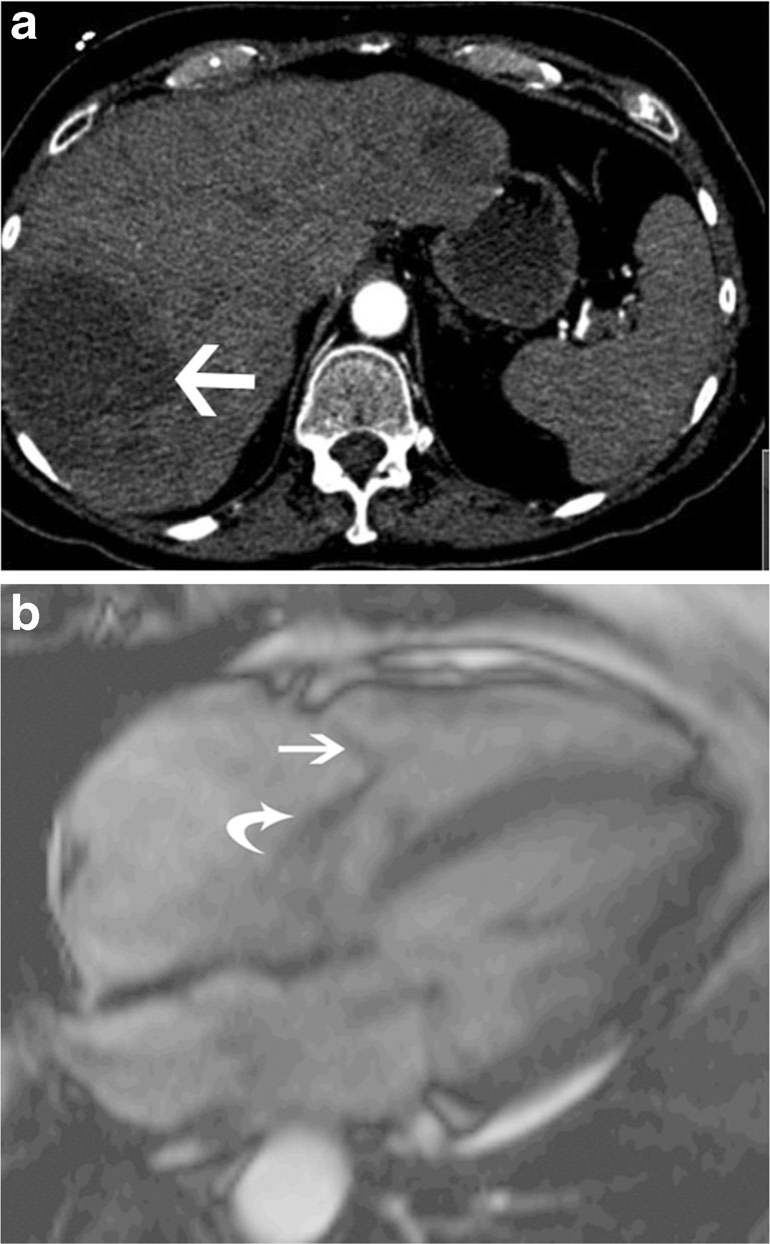
Carcinoid. (**a**) Axial CT of the abdomen shows a metastatic carcinoid tumour (*arrow*) in the liver. (**b**) Four-chamber steady-state free precession (SSFP) image shows a thickened tricuspid leaflet (*straight arrow*) with moderate tricuspid regurgitation (*curved arrow*). Mitral regurgitation is also visible

A carcinoid tumour is a slow-growing neuroendocrine cell-derived neoplasm. The appendix and terminal ileum are the most common locations of primary carcinoid tumours, whereas the liver is the most frequent site of metastasis (50 %) [24]. The tricuspid valve is the most common cardiac valve that is involved by metastatic carcinoid tumours [24]. Carcinoid syndrome occurs when vasoactive products (5-hydroxytryptamine, serotonin, histamine, tachykinins, prostaglandins) released by a metastatic liver carcinoid (Fig. 20) overwhelm the normal function of the liver to inactivate these products. Cardiac lesions are seen in 50–60 % of patients with carcinoid syndrome, typically 18–24 months after diagnosis [24].

Carcinoid plaques composed of smooth muscle cells, myofibroblasts and fibrous elastic tissue are seen on the ventricular side of the leaflets, forming a lining on the surface of the native valve, without involving the endocardium. In the early stages of carcinoid involvement, the leaflets are splayed open, resulting in regurgitation; however, with time, fibrotic reaction involving the mural wall endocardium and the valve annulus leads to thickening of the chordae and leaflets, resulting in valvular stenosis. TR is seen in 90 % of cases, eventually resulting in heart failure (Fig. 16b, Movie S3), whereas stenosis is seen in 10 % of cases. On CT and MRI, thickening of the tricuspid valvular and subvalvular apparatus is seen, with decreased motion of leaflets. Features of TR and stenosis are also seen, which are quantified as described above.

### Masses

The tricuspid valve is affected by several masses, both neoplastic and non-neoplastic. Non-neoplastic masses include thrombus, vegetation, abscess, and caseous necrosis. Benign neoplasms include fibroelastoma, myxoma, hemangioma and lipoma. Malignant neoplasms include metastasis, lymphoma and sarcomas such as angiosarcoma and leiomyosarcoma. Thrombus is the most common non-neoplastic mass, myxoma is the most common benign mass, and metastasis is the most common malignant mass.

#### Vegetations

**Fig. 21:**
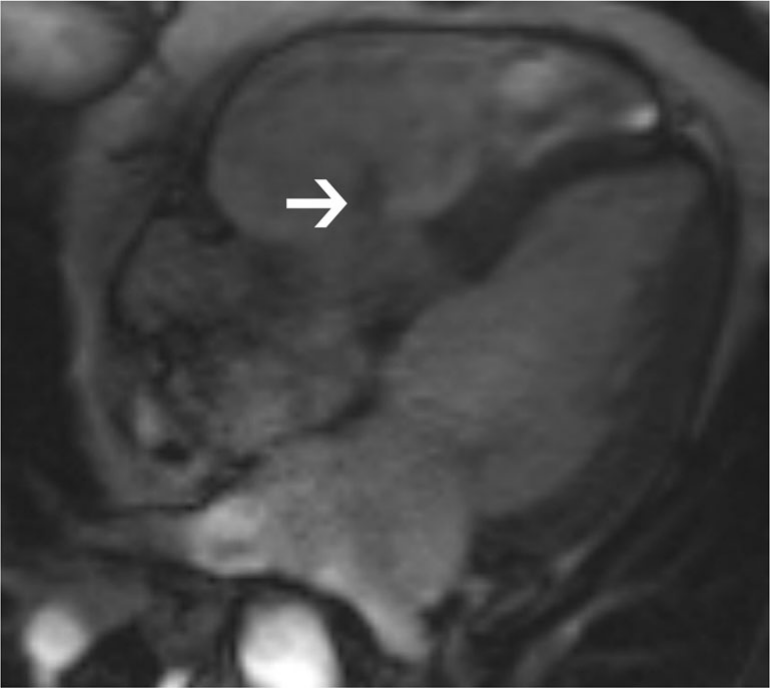
Vegetation. Four-chamber steady-state free precession (SSFP) image in a patient with septic shock and infective endocarditis shows a linear vegetation (*arrow*) attached to the septal leaflet of the tricuspid valve

Right-sided infective endocarditis (IE) is most often seen in patients with indwelling central catheters or cardiac devices, or in intravenous drug abusers [25]. Vegetations are typically located on the atrial side of the valve [26]. Accurate diagnosis of IE depends on the synthesis of presenting history, blood cultures and other laboratory work, as well as imaging findings. Echocardiography remains the primary modality for detecting vegetations and abscesses [27]), with transesophageal echocardiography (TEE) having a reported sensitivity of 85–90 %. On CT, tricuspid valve vegetation is seen as a hypoattenuating and irregular valvular mass. CT is also useful for identifying calcification and perivalvular complications such as abscesses and pseudoaneurysms. On MRI, large vegetations appear as a low-signal mass attached to a valve leaflet, with associated valvular regurgitation (Fig. 21).

#### Thrombus

**Fig. 22:**
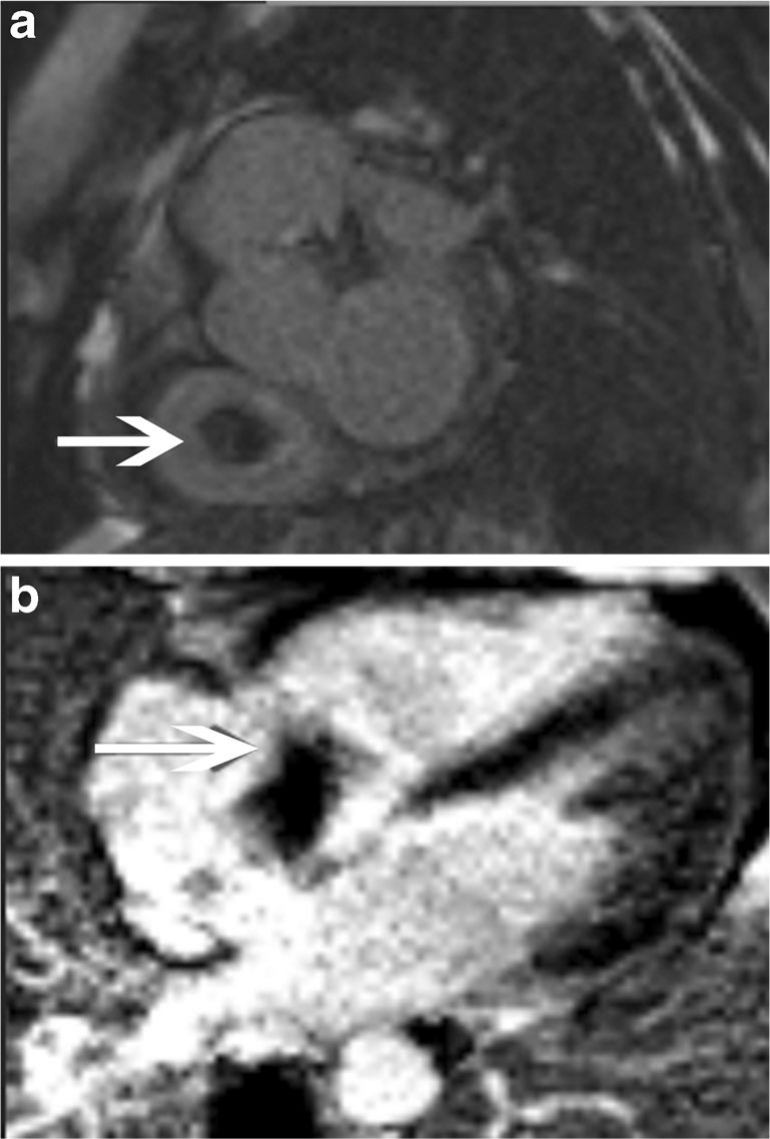
Thrombus. (**a**) Short-axis delayed enhancement (inversion time, 290 msec) shows a non-enhancing lesion attached to the tricuspid leaflet. (**b**) Four-chamber delayed-enhancement image in the same patient obtained with a longer inversion time of 600 msec shows persistent non-enhancement and low signal within the mass attached to the tricuspid valve, which is consistent with a thrombus

A thrombus in the tricuspid valve can be seen in anti-coagulable states or following placement of catheters in the RA or RV. On CT, a thrombus is seen as a non-enhancing mass attached to the tricuspid valve; on MRI, it has low signal in all sequences. Thrombi typically show no contrast enhancement in either the early or delayed phases (Fig. 22a). To distinguish a thrombus from a neoplastic mass, an additional delayed-enhancement sequence is performed at a longer inversion time (TI, 500–600 msec; Fig. 22b), in which a thrombus will have a low signal but a neoplasm will have intermediate or high signal due to contrast enhancement. A chronic thrombus, however, may vascularize and show some contrast enhancement.

#### Fibroelastoma

**Fig. 23:**
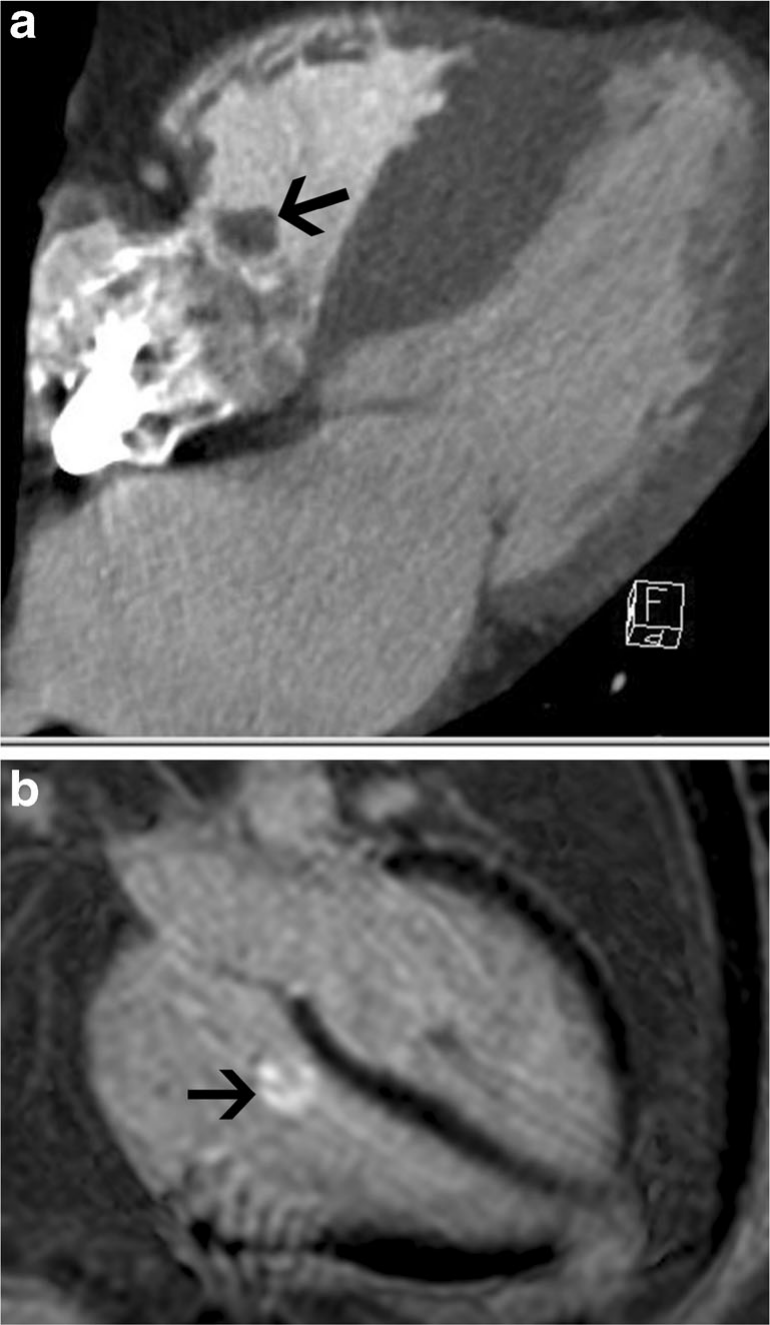
Fibroelastoma. (**a**) Four-chamber CT image shows a small, well-defined mass attached to the septal leaflet of the tricuspid valve (*arrow*), consistent with a fibroelastoma. (**b**) Four-chamber delayed-enhancement image in another patient shows heterogeneous enhancement of the tricuspid valve mass, consistent with a fibroelastoma

A fibroelastoma is the most common benign neoplasm affecting the tricuspid valve, although aortic and mitral valves are more commonly involved. Histologically, a fibroelastoma has multiple fronds of connective tissue, consisting of elastic stroma fibrous tissue, smooth muscles cells, and proteinaceous gel-like matrix covered peripherally and at the top with a loose layer of hyperplastic endocardial cells. Fibroelastomas are usually small (<5 mm), although cases of fibroelastomas up to 7 cm have been reported [28]. Typically, a fibroelastoma has a short stalk and outpouchings of long, thin, delicate fronds [29]. It is usually asymptomatic and an incidental imaging finding, but case reports of peripheral embolization have been documented. On CT and MRI, a small nodular mass is seen in the tricuspid valve, attached by a stalk (Fig. 23a). There is a homogenous intermediate T1 signal, a high T2 signal and some delayed enhancement (Fig. 23b). The sensitivity of MRI may be limited due to the small size and intermediate signal characteristics. Unlike vegetations, a fibroelastoma typically spares the free edge of valve leaflets and is likely to cause valve dysfunction.

#### Myxoma

**Fig. 24:**
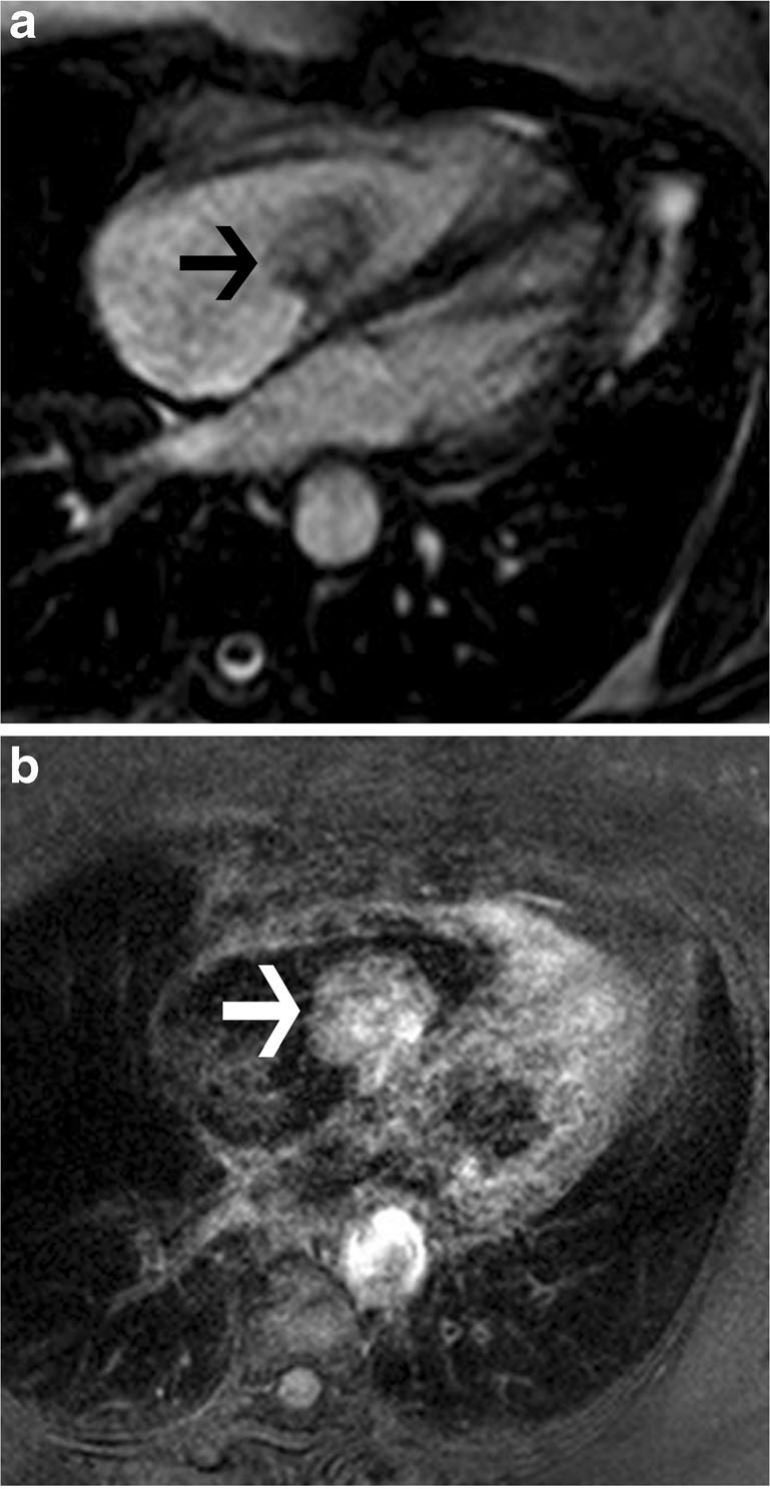
Myxoma. (**a**) Four-chamber steady-state free precession (SSFP) image shows an iso- to hypointense mass attached to the tricuspid valve. (**b**) Post-contrast T1-weighted image shows heterogeneous contrast enhancement. This was a biopsy-proven myxoma

A myxoma is composed of spindle-shaped primitive stromal cells implanted within a myxoid matrix. It has well-defined margins and is smooth, round or oval, and lobulated. Only 20 % of myxomas occur in the right heart, and even fewer originate from the tricuspid valve itself [30]. It is more common in women, especially in the 30–60-year age group [31]. Although it is a benign lesion, a myxoma can cause valvular obstruction and embolization. On CT, it is heterogenous, with foci of low attenuation and occasional calcification with patchy contrast enhancement. On MRI, a myxoma exhibits an intermediate signal on T1W images (Fig. 24a) and a high signal on T2W images. Hemorrhage has a high signal on T1. Heterogeneous early and delayed enhancement may be seen (Fig. 24b).

#### Lymphoma

**Fig. 25:**
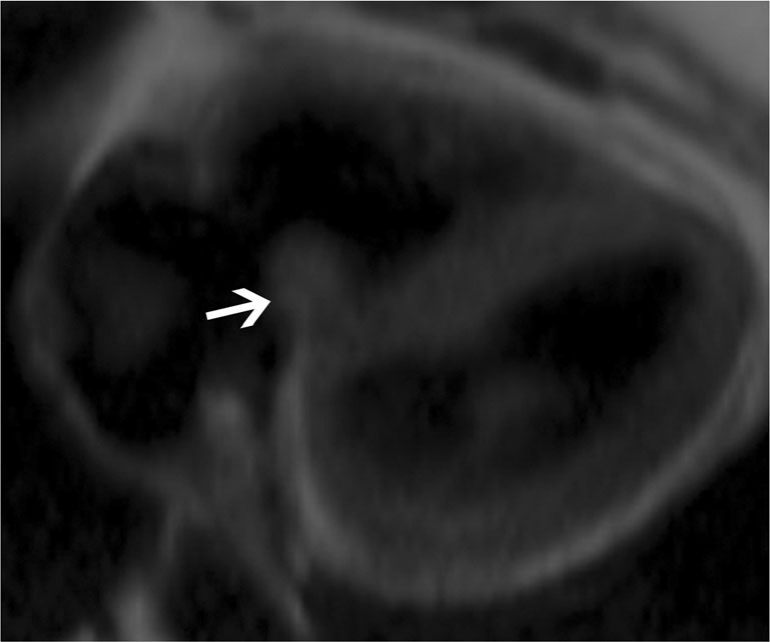
Lymphoma. Axial black-blood T2-weighted image shows a focal mass in the septal leaflet of the tricuspid valve in a patient with B-cell lymphoma

Primary cardiac lymphoma is rare, and is seen in immunocompromised patients. Secondary lymphoma is more common, and usually extends contiguously from the mediastinum or hematogenously from other systemic sites. The RA is the most commonly affected chamber (70 %) [29], although multiple chambers are usually involved. Lymphoma presents as a homogenous soft tissue mass that abuts and distorts and, less commonly, invades adjacent cardiovascular structures. Lymphoma may be seen as an infiltrative soft tissue mass, polypoid large focal mass or multiple small nodules. Pericardial effusion and mediastinal lymphadenopathy may also be seen. On MRI, tricuspid lymphoma can be isointense to slightly hypointense on T1W images, and hyperintense to isointense on T2W images (Fig. 25). Delayed enhancement is usually seen.

#### Metastases

**Fig. 26:**
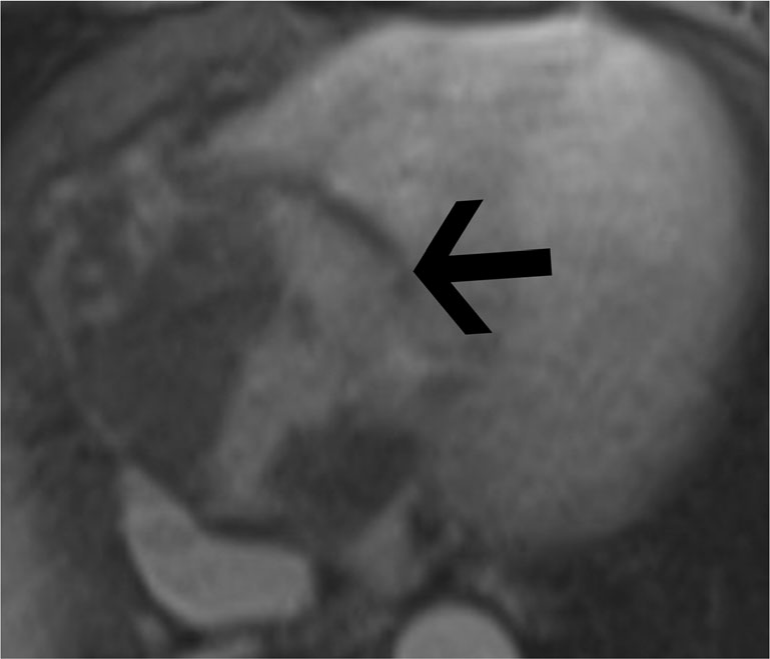
Metastasis. Axial perfusion image shows heterogeneous enhancement of a large mass (*arrow*) attached to the tricuspid valve. This is a metastasis from a primary breast carcinoma

Metastatic involvement of the tricuspid valve is much more common than any of the previously discussed primary cardiac tumours. Tricuspid valve metastasis usually indicates advanced-stage disease, and there is often evidence of other widespread systemic neoplastic foci or metastatic involvement. Metastases reach the tricuspid valve either by direct extension or by lymphatic or hematogenous spread. Cancers of the lung, leukemia/lymphoma, breast, esophagus and Kaposi sarcoma are the common primary cancers [14]. Clinically, tricuspid valve metastases may be unrecognized due to the absence of specific symptoms. On CT, metastases appear as nodular masses with irregular borders and associated myocardial thickening, disruption of the heart border, pericardial thickening and effusion. On MRI, a metastasis has a low signal on T1W images and a high signal on T2W images, with the exception of melanoma, which has high signal intensity on both T1W and T2W images. Heterogeneous contrast enhancement is seen (Fig. 26).

#### Angiosarcoma

**Fig. 27:**
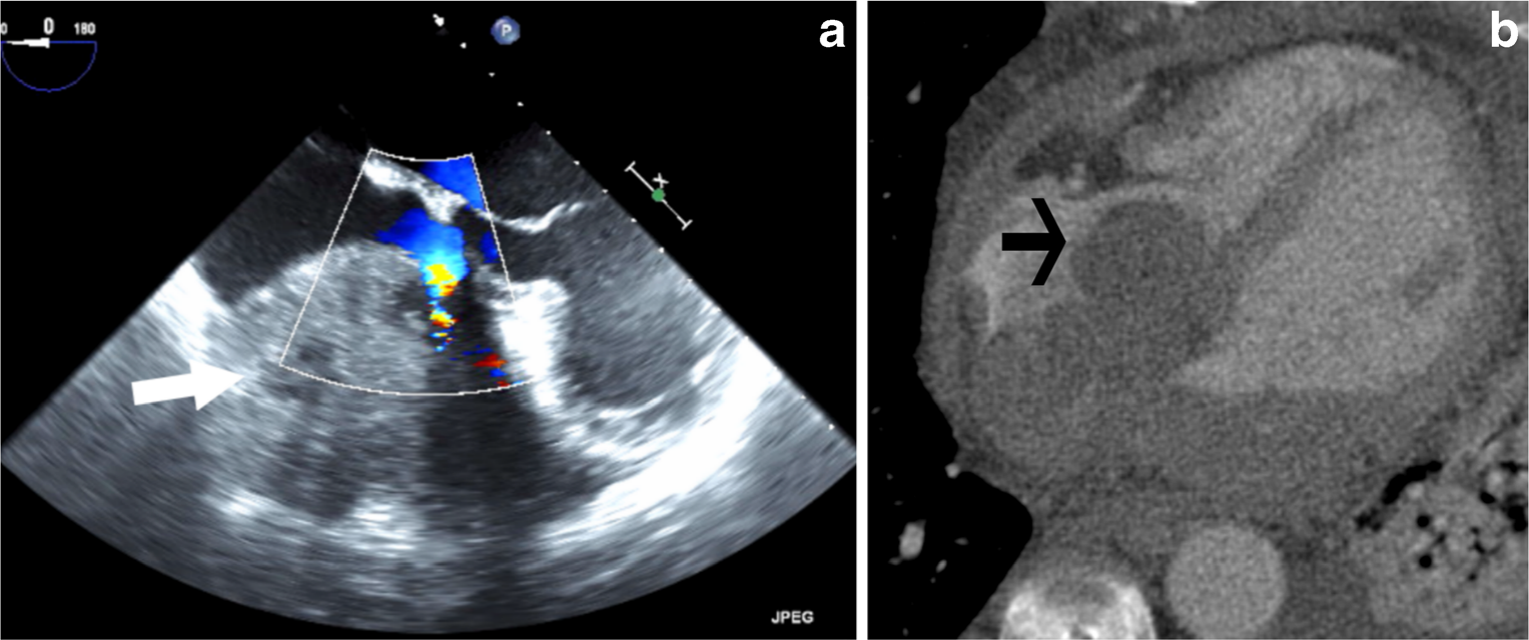
Angiosarcoma. (**a**) Echocardiogram shows a vascular tumour located in the right atrium (RA) (*arrow*), with displacement of the tricuspid leaflet and flow acceleration in the tricuspid valve. (**b**) Axial contrast-enhanced CT image shows a broad-based mass arising from the tricuspid valve (*arrow*), with involvement of the RA, interatrial septum and left atrium, and extension outside the heart into the pericardium

Angiosarcoma is the most common primary cardiac malignancy, with 75 % originating from the RA. Tricuspid valve involvement is not uncommon [32], and is more often observed in middle-aged men. Sixty percent of these cases present as multiple tumours [33]. Hemorrhagic pericardial effusion, tamponade and heart failure are common clinical features. On CT and MRI, angiosarcoma is seen as a broad-based, irregularly shaped infiltrative nodular mass, usually within the RA, with or without tricuspid valve involvement (Fig. 27). On MRI, it has low to intermediate signal intensity on T1W images, but can have a high signal due to hemorrhage. Heterogeneous signals are seen in T2W images. A heterogeneous mosaic pattern of enhancement is seen due to hemorrhage and necrosis. Infiltration of adjacent structures and distal metastasis is often seen [34].

### Tricuspid valve prolapse

Tricuspid valve prolapse is characterized by posterior bulging of the leaflets of the tricuspid valve beyond its annulus, into the RA in systole, due to sufficient elongation of the chordae and expansion of the cusp area. It is less common than mitral valve prolapse, occurs in an older age group, and may be a marker of diffuse connective tissue diseases. It is associated with mitral valve prolapse in 5–52 % of cases [35], and these patients are older and more symptomatic. Pathologically, the leaflets are tortuous and thickened, with myxomatous degeneration, loss of fibrous tissue, fragmentation and coiling of collagen bundles.

**Fig. 28:**
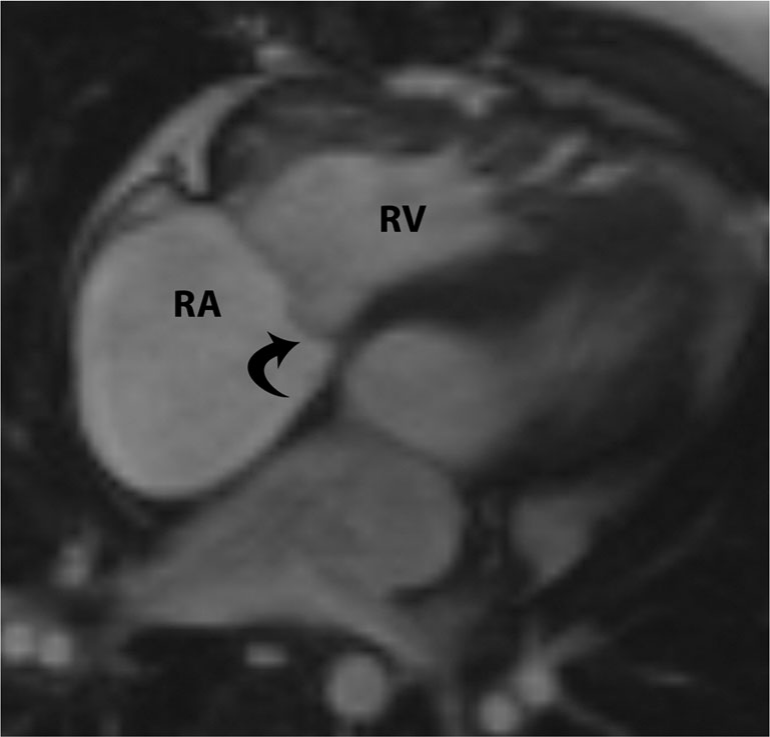
Tricuspid prolapse. Four-chamber steady-state free precession (SSFP) image shows posterior bulging of the septal leaflet into the right atrium during systole (*curved arrow*), consistent with tricuspid valve prolapse. Note that the anterior leaflet is located in normal position

On CT and MRI, the prolapse of the tricuspid valve leaflet beyond the annular ring into the RA is seen during late systole (Fig. 28). This may present a challenge, since the annular ring is non-planar, and accurate localization is difficult. An RV long-axis view is useful for identification. Tricuspid valve prolapse more commonly affects the anterior and septal leaflets, and TR is seen in 40 % of cases, occurring in early systole, before the maximum prolapse [36]. Other features are: redundant and thick leaflets; eccentric coaptation of leaflets; annular dilation; decreased change of annular size between diastole and systole and enlarged RA. Tricuspid valve prolapse is treated with annuloplasty.

### Amyloidosis

**Fig. 29:**
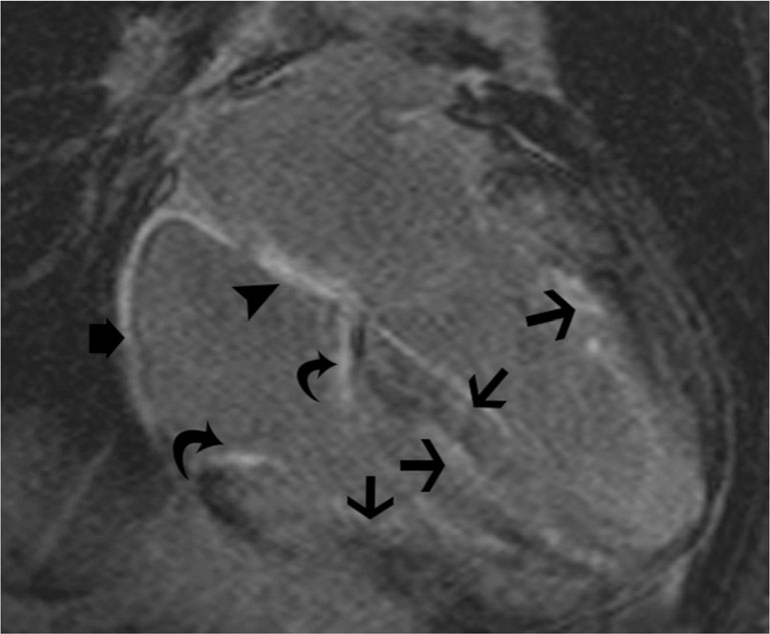
Amyloid. Four-chamber delayed-enhancement image shows delayed enhancement of the tricuspid leaflets (*curved arrows*) consistent with cardiac amyloidosis. Note also the diffuse hyperenhancement of the left and right ventricular myocardium (*straight arrows*), interatrial septum (*arrowhead*) and the right atrial wall (*short arrow*)

Cardiac amyloidosis is characterized by deposition of a ß-pleated glycoprotein. Along with infiltration of the rest of the myocardium, the tricuspid valve may also be affected. There are several types of amyloidosis, including primary, secondary, senile, AA, AL, and transthyretin types. The amyloidopathy that primarily affects the heart and the tricuspid valve usually is of the primary form (AL amyloidosis), which is seen with multiple myeloma and other monoclonal gammopathies. The interstitial deposition of proteinaceous material causes a tight ring-like band impeding myocardial fibre shortening, leading to decreased ventricular compliance, thickening of the ventricular wall, diastolic heart failure, restrictive cardiomyopathy and eventual atrophy and systolic failure. On MRI, the valve leaflets are thickened, with a diffuse low signal on T1W and T2W images. There is intense late gadolinium enhancement of the leaflets (Fig. 29), along with thickening and enhancement of the ventricular myocardium, atria and interatrial septum. Late gadolinium enhancement of the myocardium is seen often in a diffuse subendocardial or transmural pattern, but may be occasionally patchy. T1 kinetics is altered with nulling of the myocardium occurring before the blood pool. T1 mapping may also be used for quantifying the extent of cardiac amyloidosis.

## Conclusion

Although echocardiography is the most frequently used modality for the evaluation of tricuspid valve disorders, CT and MRI are also increasingly used. MRI provides not only morphological information, but also functional information on the quantification of tricuspid valvular disorders as well the secondary consequences on the ventricles.

## Electronic supplementary material

Below is the link to the electronic supplementary material.

Movie S1 Four-chamber cine-SSFP image in a patient with Ebstein’s anomaly shows a significantly apically displaced septal leaflet of the tricuspid valve, with resultant atrialization of the right ventricle and severe tricuspid regurgitation. There is mild global systolic dysfunction of the right ventricle. Note the diastolic septal bounce due to the volume load of tricuspid regurgitation. (AVI 9834 kb)

Movie S2 Gerbode defect. Four-chamber cine-SSFP image shows a Gerbode defect resulting in shunting between the left ventricle and right atrium. (AVI 136329 kb)

Movie S3 Four-chamber cine-SSFP shows thickening of the tricuspid and mitral leaflets with associated tricuspid and mitral regurgitation in a patient with a carcinoid tumor involving all the leaflets. (AVI 25018 kb)
